# A study on a real-world data-based VTE risk prediction model for lymphoma patients

**DOI:** 10.3389/fphar.2025.1691271

**Published:** 2025-10-14

**Authors:** Changli He, Yin Wang, Han Zhang, Sitian Li, Fengjiao Kang, Fengqun Cai, Lizhu Han, Qinan Yin, Gang Li, Xuewu Song, Yuan Bian

**Affiliations:** ^1^ Department of Pharmacy, Personalized Drug Research and Therapy Key Laboratory of Sichuan Province, Sichuan Provincial People’s Hospital, School of Medicine, University of Electronic Science and Technology of China, Chengdu, China; ^2^ Pharmacy Department of Xinjiang Medical University Affiliated Traditional Chinese Medicine Hospital, Urumqi, Xinjiang, China

**Keywords:** lymphoma, venous thromboembolism, machine learning, predictive factors, predictive model

## Abstract

**Background:**

Patients diagnosed with malignant tumors exhibit a markedly elevated risk of venous thromboembolism (VTE), which has a negative impact on their prognosis. Currently, there is no reliable predictive model specifically for thrombosis risk in lymphoma patients. This study aims to develop and validate a machine learning model leveraging real-world data, offering a dependable risk assessment tool for the early identification of VTE in lymphoma patients.

**Methods:**

We retrospectively analyzed 605 hospitalized patients with lymphoma between January 2019 and June 2024. Candidate predictors included demographic characteristics, comorbidities and medical history, tumor-related factors, treatment-related factors, and laboratory parameters. The primary endpoint was the occurrence of VTE within 6 months after hospitalization for confirmed lymphoma. Model development incorporated three imputation methods, three sampling strategies, three feature selection approaches, and nine machine learning algorithms. Predictive performance was compared across all models.

**Results:**

Combining different imputation, sampling, and feature selection strategies yielded 27 datasets, which were trained across nine algorithms to generate 243 models. The optimal model—Simp-SMOTE_rf_GBM, constructed using random forest imputation, SMOTE oversampling, and gradient boosting machine—achieved the highest predictive performance (AUC = 0.954). SHAP-based model interpretation identified nine key predictors ranked by importance: anticoagulant use, D-dimer, lactate dehydrogenase, central venous catheterization, carcinoembryonic antigen (CEA), Eastern Cooperative Oncology Group (ECOG) score, serum total protein (TP), total cholesterol (TC), and infectious disease.

**Conclusion:**

This study established and validated a machine learning model for predicting VTE risk in lymphoma patients, with the optimal model demonstrating excellent discriminatory ability (AUC = 0.954). The model provides evidence to guide the timing and strategy of anticoagulation, supporting early VTE screening and risk stratification in clinical practice. Its implementation has important implications for improving patient outcomes and advancing public health.

## Introduction

Cancer is one of the leading causes of global disease burden, accounting for approximately one-sixth of all deaths worldwide (Bray et al., 2021; 2024). Beyond its impact on health, cancer imposes a substantial economic burden and has become a major global public health concern (Chen et al., 2023). Within the spectrum of hematological malignancies, lymphoma exhibits the highest incidence globally (Ying X. H et al., 2024). Lymphoma comprises a heterogeneous group of malignancies arising from the lymphoid system and the potential to involve multiple anatomical sites, including lymph nodes, tonsils, spleen, and bone marrow (Bobillo et al., 2022). Recent epidemiological trends reveal a concerning 5% annual increase in lymphoma incidence worldwide (Meng, 2019). The 2020 Global Cancer Statistics Report documented approximately 630,000 incident lymphoma cases globally, with projections suggesting this burden will escalate to 910,000 cases by 2040 (Sung et al., 2021). In China, the incidence of lymphoma is also growing rapidly, ranking eighth among all cancer types (National Cancer Center, 2025).

VTE, comprising deep vein thrombosis (DVT) and pulmonary embolism (PE), is a common complication and a leading cause of mortality among hospitalized patients (Khan et al., 2013). Epidemiological data indicate that adult cancer patients face a 4 to 6.5-fold higher risk of VTE compared with noncancer populations (Kekre and Connors, 2019). Hematologic malignancies confer an even greater thrombotic risk than solid tumors (Blom et al., 2005), with lymphoma patients particularly predisposed to VTE, a risk that continues to rise annually (Wan, 2024). Reported incidence rates of VTE in lymphoma range from 5% to 17% (Sanfilippo et al., 2016). Notably, non-Hodgkin lymphoma (NHL) carries a higher thrombotic risk than Hodgkin lymphoma (HL). Mohren et al. reported a VTE incidence of 10.6% among patients with high-grade NHL, compared with 7.65% in HL and 5.8% in low-grade NHL (Mohren et al., 2005). A meta-analysis by Caruso et al. further confirmed this difference, with a thrombosis incidence rate of 6.5% in NHL patients and only 4.7% in HL patients (P < 0.001) (Caruso et al., 2010). Within NHL subtypes, difference persists: in a U.S. single-center retrospective study, the 1- and 5-year incidence of VTE in follicular lymphoma was 2.4% and 3.8%, respectively, markedly lower than 10.8% and 16.3% observed in patients with diffuse large B-cell lymphoma (DLBCL) (Dharmavaram et al., 2020). The occurrence of VTE not only leads to limb pain, impaired mobility, and reduced quality of life but also disrupts chemotherapy and is associated with inferior survival outcomes.

Although prophylactic anticoagulation can effectively prevent VTE and recurrence, it also increases the risk of bleeding. In contrast to solid malignancies, lymphoma exhibits greater bone marrow invasiveness, frequently resulting in thrombocytopenia and consequent bleeding diathesis, further exacerbating the economic and clinical burden (Shang et al., 2023). Consequently, achieving optimal risk-benefit equilibrium with prophylactic anticoagulation remains a pressing clinical dilemma and therapeutic challenge. International guidelines emphasize the need for accurate and efficient VTE risk assessment tools to identify high-risk patients and to inform tailored prevention and management strategies (Streiff et al., 2021).

## Methods

### Study population

We performed a retrospective cohort analysis of 605 lymphoma patients admitted to Sichuan Provincial People’s Hospital between January 2019 and June 2024. Inclusion criteria comprised: (1) Age ≥18 years; (2) Histopathologically confirmed lymphoma diagnosis according to the 2022 WHO Classification of Hematopoietic and Lymphoid Tumors (WHO-HAEM5) criteria. Exclusion criteria included: (1) Prior anticancer therapy at external institutions; (2) Secondary lymphoma or concurrent multiple primary malignancies; (3) VTE events diagnosed before lymphoma confirmation; (4) Incomplete hospitalization records; (5) Insufficient follow-up (<6 months) for VTE assessment. VTE occurrence within 6 months was ascertained through comprehensive review of electronic medical records (EMR), including inpatient documentation, outpatient visits, and confirmatory imaging studies (e.g., compression ultrasonography). The study protocol received approval from the Institutional Review Board of Sichuan Provincial People’s Hospital [Ethics Review (Research) No. 526 of 2024].

### Data collection

Potential VTE-associated predictors were identified through a systematic literature review and expert consultation. Clinical data were extracted retrospectively from the hospital’s EMR system. (1) Demographics: age, height, weight, Body mass index (BMI), sex, smoking status, chronic alcohol use (>5 years), and Eastern Cooperative Oncology Group (ECOG) score. (2) Comorbidities and medical history: hypertension, diabetes mellitus, active infections, hepatic disorders, electrolyte disturbances, pulmonary comorbidities, and prior transfusion history; (3) Tumor-related factors: tumor histological subtype, tumor stage, recurrence or refractory lymphoma, extranodal involvement, mediastinal involvement, bone marrow involvement, central nervous system involvement, splenic involvement, B symptoms, large mass (>10 cm); (4) Treatment-related factors: platinum-based drugs, anthracycline-based drugs, rituximab, erythropoietin/granulocyte colony-stimulating factor, etc.; (5) Laboratory parameters: D-dimer, Prothrombin time (PT), activated partial thromboplastin time (aPTT), fibrinogen, white blood cell count (WBC), platelets, hemoglobin, neutrophils, monocytes, hypersensitive C-reactive protein (hs-CRP), erythrocyte sedimentation rate (ESR), etc. For patients with multiple admissions, only baseline data from the index hospitalization were analyzed. All patient identifiers were anonymized and replaced with unique study identification codes.

### Data preprocessing

Because of missing data, class imbalance, and the high dimensionality of candidate predictors, data preprocessing included imputation, resampling, and feature selection to reduce the risk of overfitting and the “curse of dimensionality.” Comprehensive data preprocessing was through three key approaches: (1) Data imputation: K-nearest neighbors (KNN), random forest, and predictive mean matching; (2) Data sampling: random oversampling, SMOTE (Synthetic Minority Over-sampling Technique), and Borderline-SMOTE; (3) Feature selection: LASSO (Least Absolute Shrinkage and Selection Operator), ridge, and elastic net regression. Through a full factorial combination of these methods, we generated 27 distinct processed datasets for subsequent model development. Data cleaning methods and algorithm ID assignments are shown in Supplementary Table S1.

### Model development and evaluation

Datasets were randomly split into training (80%) and test (20%) sets with stratified sampling to maintain outcome distribution. The training set facilitated model development using nine distinct machine learning (ML) algorithms, with the test set reserved for independent performance evaluation. The ML algorithms include logistic regression (LR), decision trees (DT), random forests (RF), support vector machines (SVM), naive Bayes (NB), KNN, gradient boosting machine (GBM), extreme gradient boosting (XGBoost), and adaptive boosting (AdaBoost). We employed the training set for model construction and utilized a 10-fold cross-validation approach coupled with a grid search strategy to optimize the parameters of the top-performing machine learning algorithm on the training set. Subsequently, feature selection was conducted using all available variables, followed by model rebuilding. Next, simplify the model by reducing the number of feature variables. The top nine ranked predictors were then used to rebuild simplified models with the five highest-performing algorithms, followed by hyperparameter optimization. This step assessed the trade-off between parsimony and predictive performance.

A comprehensive evaluation framework was employed to assess model performance across three critical dimensions: discrimination, calibration, and clinical utility. Discrimination was quantified using multiple metrics: accuracy, specificity, sensitivity (recall), positive predictive values (PPV), negative predictive values (NPV), F1-score, and area under the curve (AUC). The calculation formulas for evaluation metrics are provided in Supplementary Table S2. Clinical net benefit was rigorously evaluated through decision curve analysis (DCA) across clinically relevant probability thresholds. Model interpretability was achieved using Shapley Additive exPlanations (SHAP) analysis implemented in R (version 4.2.1). Feature importance was systematically ranked based on mean absolute SHAP values to identify the most influential predictors.

### Statistical analysis

To ensure data integrity, dual independent data entry with cross-verification was performed by trained research assistants, followed by systematic quality control checks. Variables exceeding 80% missing data or exhibiting extreme outliers were excluded during preprocessing. Initial univariate analyses compared baseline characteristics between VTE and non-VTE cohorts to identify potential associations. Normally distributed continuous variables were expressed as mean ± standard deviation and compared using t-tests. Quantitative variables that did not follow a normal distribution were expressed as median and interquartile range and analyzed using the Kruskal–Wallis test. Categorical variables were presented as counts (percentages) and analyzed using Pearson’s χ^2^ or Fisher’s exact tests, as appropriate for expected cell frequencies. A two-sided α level of 0.05 defined statistical significance for all analyses. All data were analyzed using SPSS Statistics 26.0 software and R statistical software (version 4.0.3; https://www.r-project.org).

## Result

### Patient population characteristics

**FIGURE 1:**
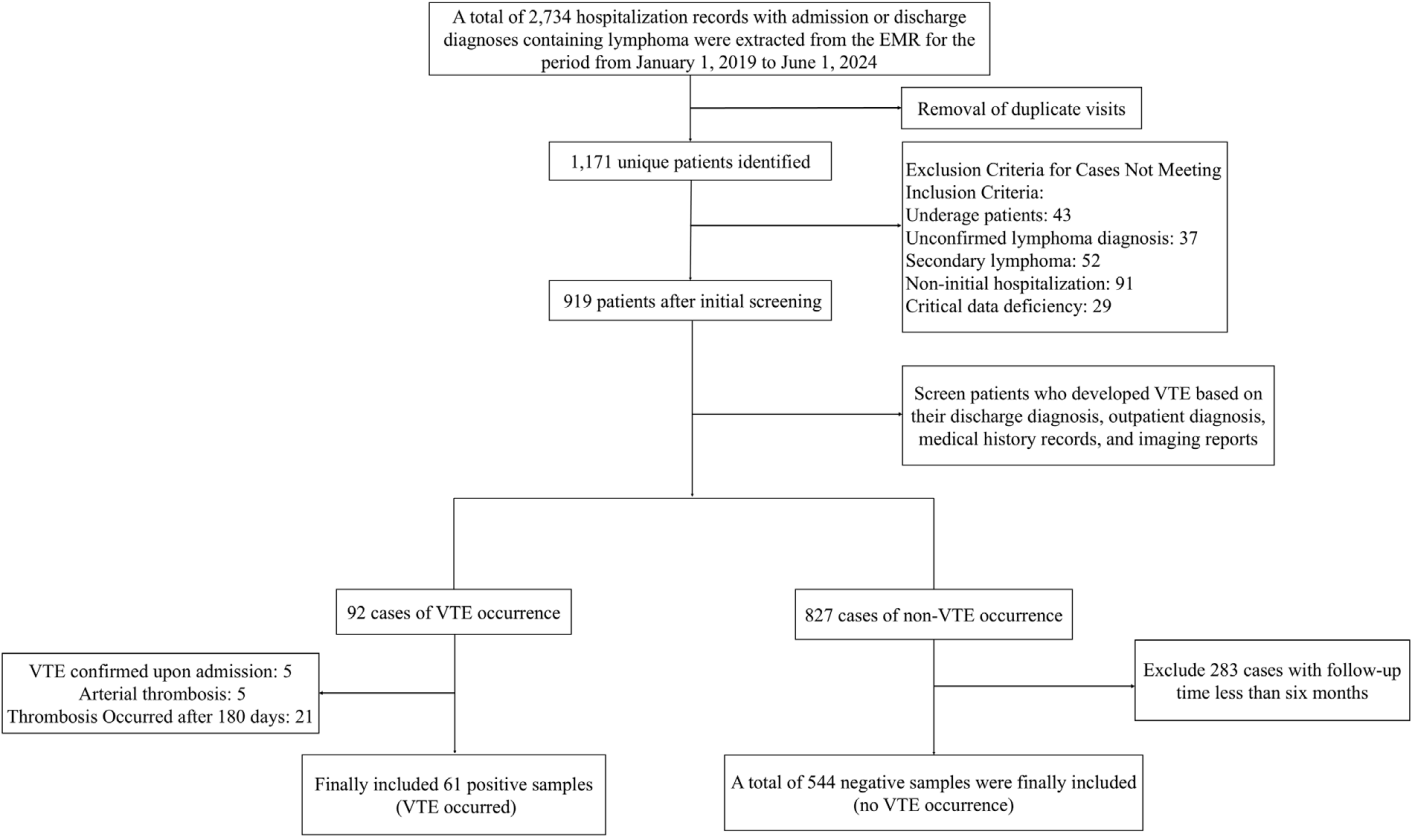
Patient screening flowchart.

A total of 2,734 hospitalization records of patients with lymphoma were retrieved from the EMR system. After de-duplication, 1,171 valid records remained. The final analytic cohort comprised 605 eligible patients meeting all inclusion criteria. The cohort included 61 VTE cases (incidence 10.1%) and 544 matched controls. Figure 1 presents the patient selection process.

The final cohort comprised 605 patients (277 female [45.8%]; 328 male [54.2%]) with a mean age of 55.5 ± 14.5 years. Regarding histological classification: Aggressive NHL predominated (n = 410, 67.8%), followed by indolent NHL (n = 159, 26.3%) and HL (n = 25, 4.1%). Of patients with documented staging (n = 564), most presented with advanced disease (n = 367, 65.1%). Central venous access was utilized in 38.8% (n = 235) of cases. Complete baseline characteristics are summarized in Table 1.

**TABLE 1:** Baseline characteristics of patients.

Variable name	Subclassification	Count	Proportion (%)
Sex			
	Female	277	45.8%
	Male	328	54.2%
Ethnicity			
	Han Chinese	580	95.9%
	Ethnic Minority	25	4.1%
Smoking History			
	Yes	167	27.6%
	No	438	72.4%
Chronic Alcohol Use			
	Yes	85	14.0%
	No	520	86.0%
ECOG score			
	<2	74	12.2%
	≥2	257	42.5%
	Not Specified	274	45.3%
Atherosclerosis			
	Yes	49	8.1%
	No	556	91.9%
Hypertension			
	Yes	115	19.0%
	No	490	81.0%
Diabetes Mellitus			
	Yes	72	11.9%
	No	533	88.1%
Atrial Fibrillation			
	Yes	9	1.5%
	No	596	98.5%
Cardiac Insufficiency			
	Yes	21	3.5%
	No	584	96.5%
Cerebral/Myocardial Infarction			
	Yes	30	5.0%
	No	575	95.0%
Hyperlipidemia			
	Yes	17	2.8%
	No	588	97.2%
Hyperuricemia			
	Yes	53	8.8%
	No	552	91.2%
Infectious Diseases			
	Yes	185	30.6%
	No	420	69.4%
Autoimmune Diseases			
	Yes	11	1.8%
	No	594	98.2%
Hepatic Disorders			
	Yes	181	29.9%
	No	424	70.1%
Renal Disorders			
	Yes	33	5.5%
	No	572	94.5%
Electrolyte Imbalance			
	Yes	113	18.7%
	No	492	81.3%
Acute Intoxication			
	Yes	9	1.5%
	No	596	98.5%
Pulmonary Diseases			
	Yes	81	13.4%
	No	524	86.6%
Hemorrhage			
	Yes	26	4.3%
	No	579	95.7%
Transfusion History			
	Yes	69	11.4%
	No	536	88.6%
Recent Surgery/Trauma (≤1 month)			
	Yes	14	2.3%
	No	591	97.7%
VTE History			
	Yes	5	0.8%
	No	600	99.2%
Histological Subtype			
	Hodgkin Lymphoma	25	4.1%
	Aggressive Lymphoma	410	67.8%
	Indolent Lymphoma	159	26.3%
	Not Specified	11	1.8%
Ann Arbor Stage			
	Stage I-II	197	32.6%
	Stage III-IV	367	60.7%
	Not Specified	41	6.7%
Relapsed/Refractory Lymphoma			
	Yes	24	4.0%
	No	581	96.0%
Extranodal Involvement			
	Present	413	68.3%
	Absent	157	26.0%
	Not Specified	35	5.8%
Mediastinal Involvement			
	Present	177	29.3%
	Absent	396	65.5%
	Not Specified	32	5.3%
Bone Marrow Involvement			
	Present	166	27.4%
	Absent	385	63.6%
	Not Specified	54	8.9%
CNS Involvement			
	Present	23	3.8%
	Absent	581	96.0%
	Not Specified	1	0.2%
Splenic Involvement			
	Present	140	23.1%
	Absent	448	74.1%
	Not Specified	17	2.8%
Bulky Disease (>10 cm)			
	Present	89	14.7%
	Absent	504	83.3%
	Not Specified	12	2.0%
B Symptoms			
	Present	190	31.4%
	Absent	394	65.1%
	Not Specified	21	3.5%
Platinum-based Agents			
	Received	75	12.4%
	Not Received	530	87.6%
Glucocorticoids			
	Received	586	96.9%
	Not Received	19	3.1%
Gemcitabine			
	Received	49	8.1%
	Not Received	556	91.9%
Thalidomide/Lenalidomide			
	Received	30	5.0%
	Not Received	575	95.0%
Anthracyclines			
	Received	408	67.4%
	Not Received	197	32.6%
Rituximab			
	Received	429	70.9%
	Not Received	176	29.1%
ESAs/G-CSF			
	Received	292	48.3%
	Not Received	343	56.7%
Methotrexate			
	Received	118	19.5%
	Not Received	487	80.5%
Cyclophosphamide			
	Received	412	68.1%
	Not Received	193	31.9%
Amphotericin B			
	Received	21	3.5%
	Not Received	584	96.5%
Anticoagulants			
	Received	221	36.5%
	Not Received	384	63.5%
Antiplatelet Agents			
	Received	47	7.8%
	Not Received	558	92.2%
Hemostatic Agents			
	Received	49	8.1%
	Not Received	556	91.9%
Venous Catheterization			
	Performed	235	38.8%
	Not Performed	370	61.2%
General Anesthesia Surgery			
	Performed	129	21.3%
	Not Performed	476	78.7%

ECOG, Eastern Cooperative Oncology Group; VTE, Venous thromboembolism; CNS, central nervous system; ESAs/G-CSF, erythropoiesis-stimulating/granulocyte colony-stimulating factors.

Of the 61 patients with lymphoma complicated by VTE, 83.6% (n = 51) had aggressive histology. The VTE incidence peaked at 45.9% (28/61) by 30 days post-diagnosis, with subsequent rates of 27.9% at 90 days and 26.2% at 180 days. Advanced-stage disease (III-IV) conferred a higher VTE risk (70.5% vs. 18.0% in early-stage). VTE manifestations included: lower extremity DVT (49.2%, n = 30), upper extremity DVT (18.0%, n = 11), cervical venous thrombosis (9.8%, n = 6), PE (8.2%, n = 5), portal vein thrombosis (3.3%, n = 2), multisite thrombosis (11.5%, n = 7). Comprehensive VTE characteristics and comparative analyses are detailed in Supplementary Table S3 from the supplementary material.

### Differences between patient groups

Univariate analysis demonstrated statistically significant differences (P < 0.05) between the two groups in baseline characteristics (age, ECOG score), comorbidities (diabetes, infectious diseases, electrolyte disturbances), tumor features [histological subtype, staging, central nervous system involvement (CNS)], treatment-related factors (transfusion history, EPO/G-CSF usage, central venous access, anticoagulant use), and laboratory parameters [D-dimer, fibrin degradation products (FDP), hs-CRP, LDH, red blood cell count, hematocrit, albumin, TP, serum calcium]. Detailed data are presented in Supplementary Table S4 from supplementary material.

### Feature selection results

**FIGURE 2:**
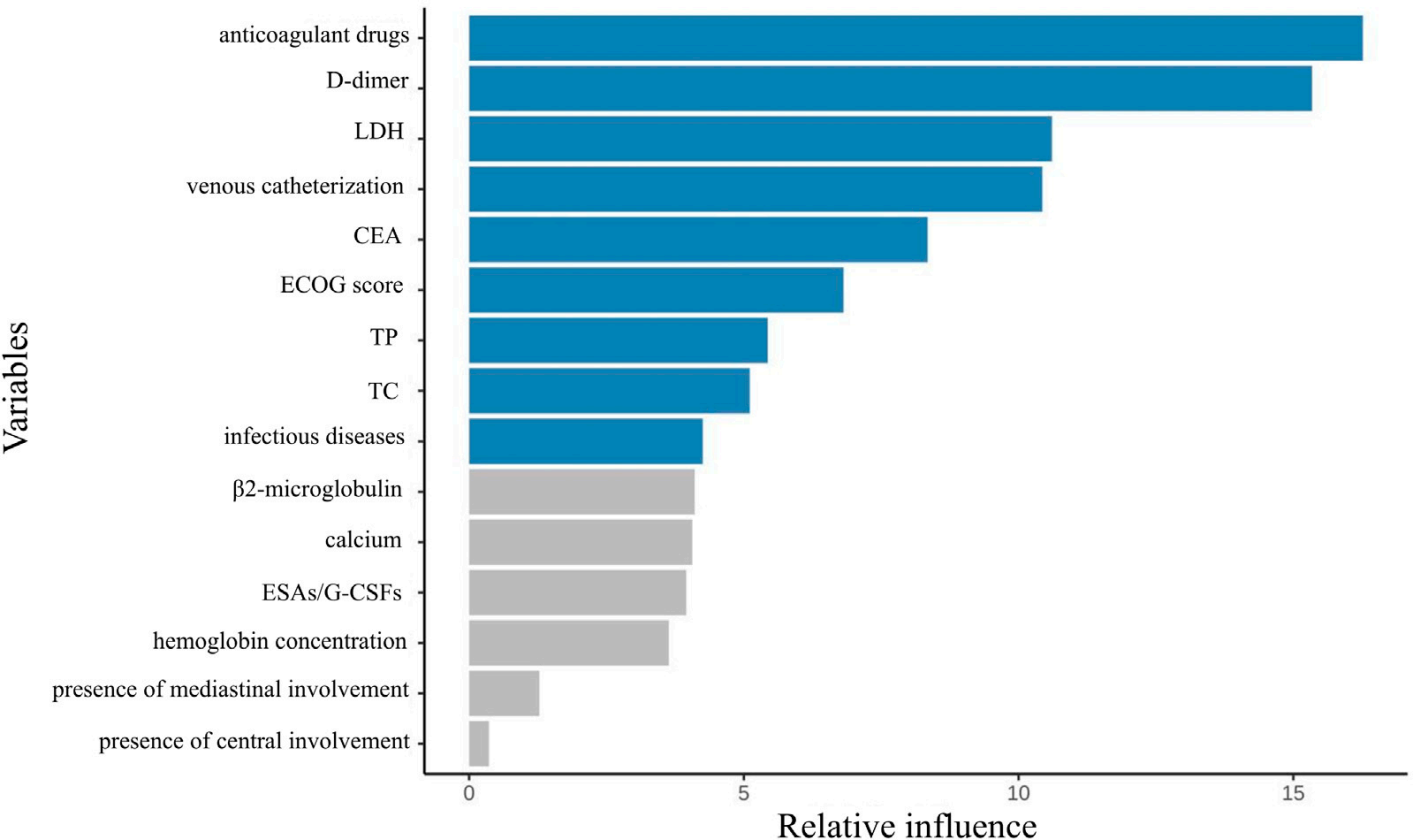
Feature importance ranking after screening based on elastic network regression.

From an initial 63 candidate variables, feature selection was performed to reduce redundancy. Since the feature dimension was still redundant after screening, the study only presented the top 15 most important variables in the performance-optimal model constructed based on all variables, with the aim of improving analysis efficiency to meet actual analysis needs. Figure 2 illustrates the feature importance ranking. The weights of these features are ranked from highest to lowest as follows: anticoagulant drugs, D-dimer, lactate dehydrogenase levels, use of intravenous catheters, CEA, ECOG score, TP, TC, infectious diseases, β2-microglobulin, calcium, erythropoiesis-stimulating/granulocyte colony-stimulating factors (ESAs/G-CSFs), hemoglobin concentration, presence of mediastinal involvement, and presence of central involvement.

### Model construction and evaluation results

**FIGURE 3:**
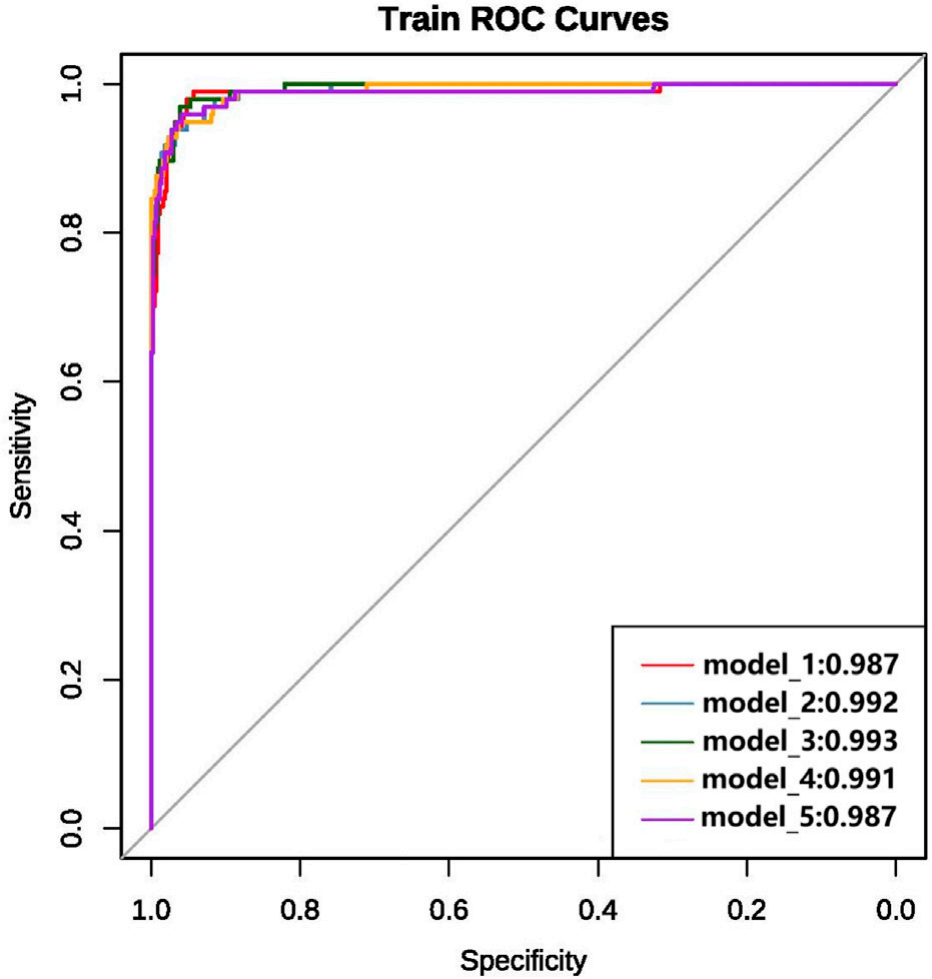
ROC curves and AUC values of the top five models on the training set after variable selection.

**FIGURE 4:**
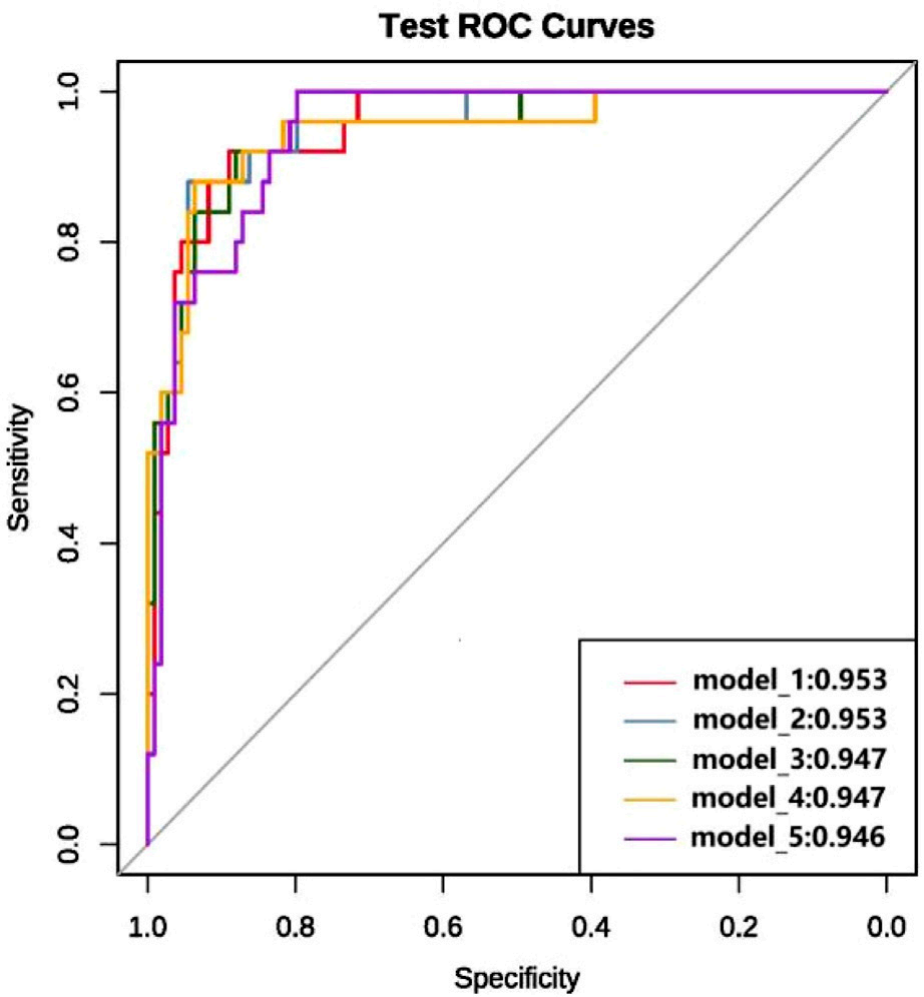
ROC curves and AUC values of the top five models in terms of modeling performance after full variable screening on the test set.

We ultimately constructed 243 models. In the training set, the five best models achieved AUCs of 0.987, 0.992, 0.993, 0.991, and 0.987, respectively (Figure 3). Performance was subsequently evaluated in the test set, with evaluation metrics for the top five models reported in Table 2 (See Supplementary Table S5 for the evaluation metrics of the other models) and their ROC curves shown in Figure 4. The optimal model combined k-nearest neighbors imputation, Synthetic Minority Over-sampling Technique (SMOTE), elastic-net–based feature selection, and a gradient boosting machine (GBM). In the test set, it achieved an AUC of 0.953 [95% confidence interval (CI): 0.932-0.974], accuracy of 0.903 (95% CI: 0.872-0.934), recall of 0.908 (95% CI: 0.864-0.952), and F1-score of 0.894 (95% CI: 0.853-0.935), significantly outperforming other approaches (P < 0.01). Feature-importance analysis identified the top nine predictors as venous catheterization, D-dimer, anticoagulant drugs, LDH, TP, β2-microglobulin, erythropoiesis or granulopoiesis-stimulating drugs, CEA, and ECOG score.

**TABLE 2:** Performance metrics of top five models selected from complete feature set.

Model name	Imputation method	Sampling method	Feature selection	Algorithm	AUC	Accuracy	Recall	Specificity	NPV	PPV	F1
model_1	0	1	2	6	0.953	0.903	0.908	0.880	0.688	0.971	0.894
model_2	1	1	0	6	0.953	0.925	0.945	0.840	0.778	0.963	0.889
model_3	2	1	2	6	0.947	0.910	0.927	0.840	0.724	0.962	0.881
model_4	1	1	2	6	0.947	0.925	0.945	0.840	0.778	0.963	0.889
model_5	0	1	0	6	0.946	0.866	0.890	0.760	0.613	0.942	0.820

**FIGURE 5:**
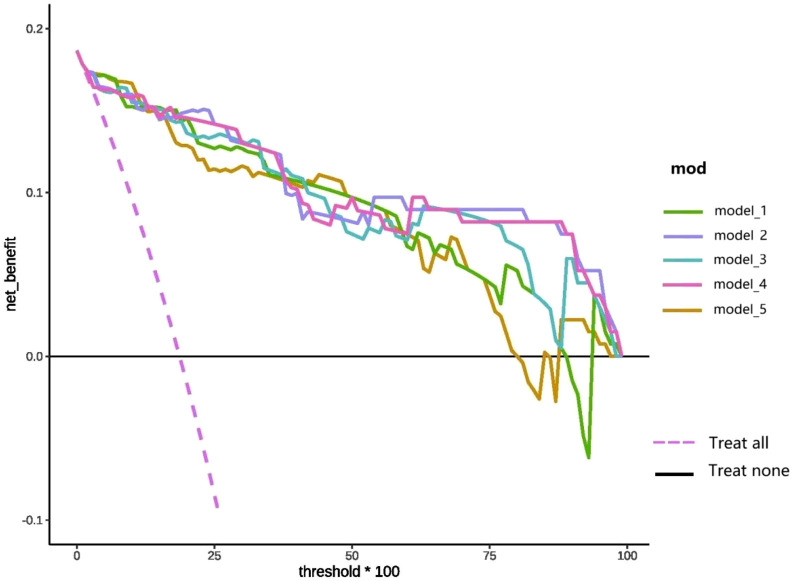
DCA curve of the top five models selected based on the entire feature set.

DCA of the top five prediction models following comprehensive feature selection is presented in Figure 5. As demonstrated in Figure 5, model_1 shows superior net benefit compared to the “treat-all” and “treat-none” reference lines across the 0%–85% probability threshold range. Similarly, when the threshold is within the 0%–75% probability range, model_5 has high clinical application value. Notably, models 2, 3, and four maintain clinical validity throughout the entire threshold spectrum (0%–100%), with models two and four consistently outperforming model three in terms of net benefit.

**FIGURE 6:**
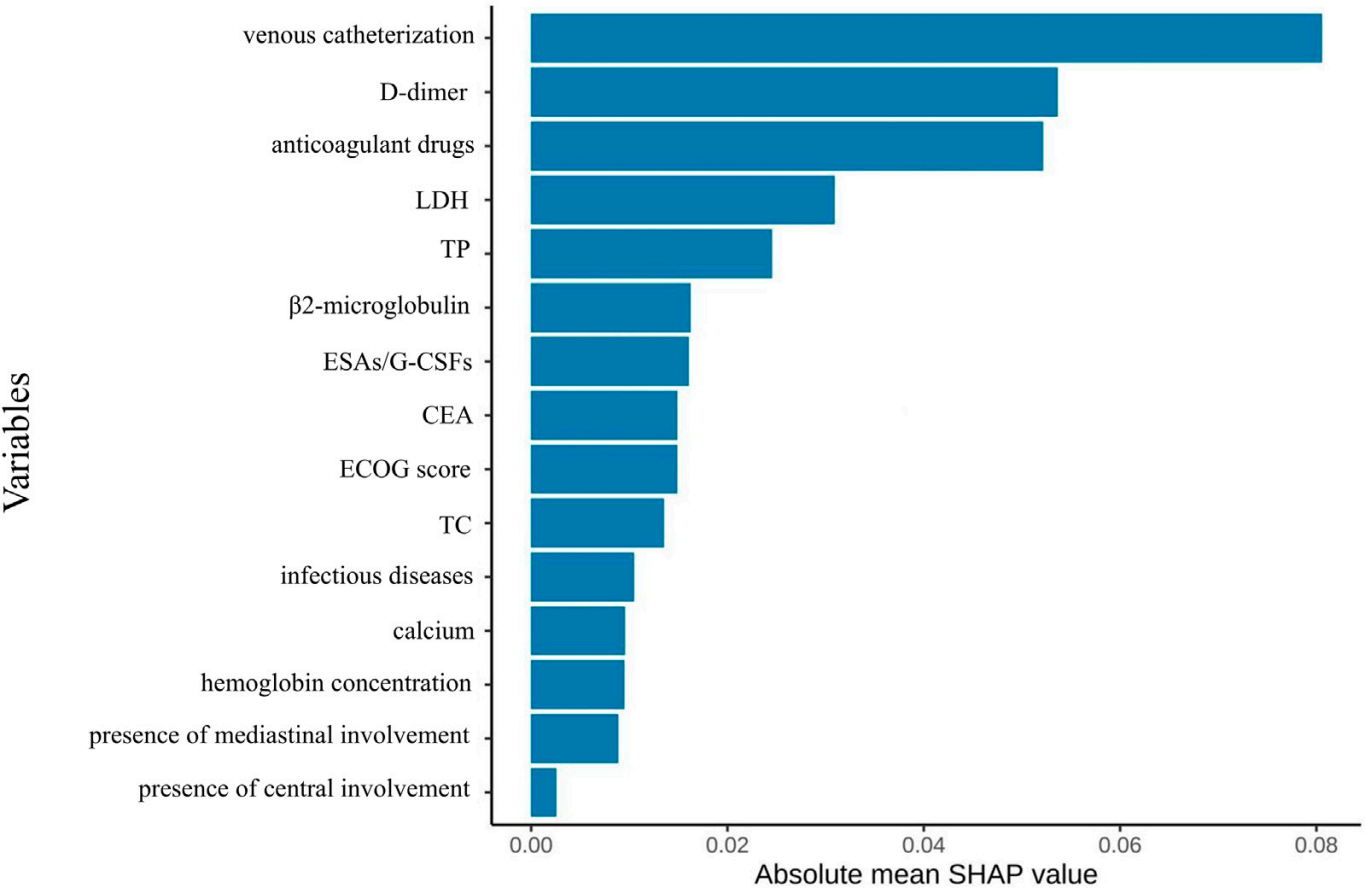
Feature importance ranking diagram of the optimal model constructed based on full variable feature selection.

We employed SHAP analysis to quantify the relative contribution of each predictive feature in the model. Figure 6 presents the SHAP summary plot for the optimal model, displaying feature importance rankings derived from comprehensive feature selection. Global interpretation revealed the mean absolute SHAP values for each feature, ranked in descending order of contribution to model predictions. Venous catheterization emerged as the most influential predictor. Subsequent predictors included: D-dimer, anticoagulant drugs, LDH, TP, etc.

**FIGURE 7:**
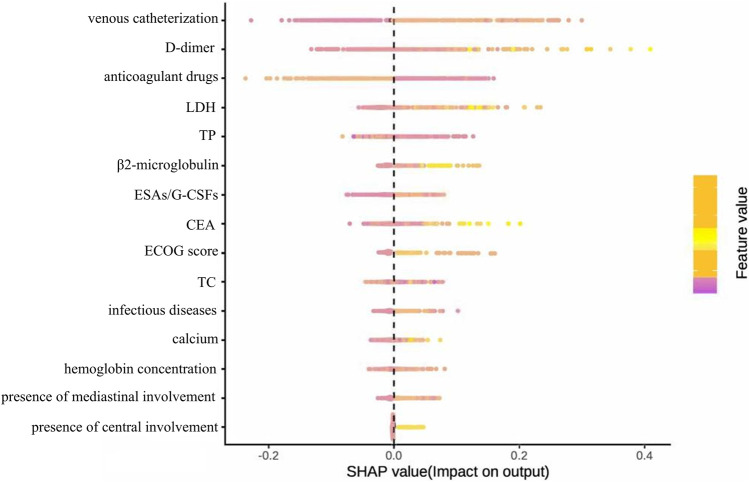
Summary diagram of the optimal model SHAP constructed based on full variable feature selection.

Figure 7 presents the SHAP beeswarm plot of the optimal model derived from comprehensive feature selection, illustrating feature importance and effect directions across the entire test cohort. Venous catheterization had the strongest positive association with VTE risk, followed by D-dimer, anticoagulant use, LDH, and TP. Preventive anticoagulation, TP, and cholesterol were negatively associated with VTE, while mediastinal and CNS involvement showed weak predictive value.

### Simplified model results

**FIGURE 8:**
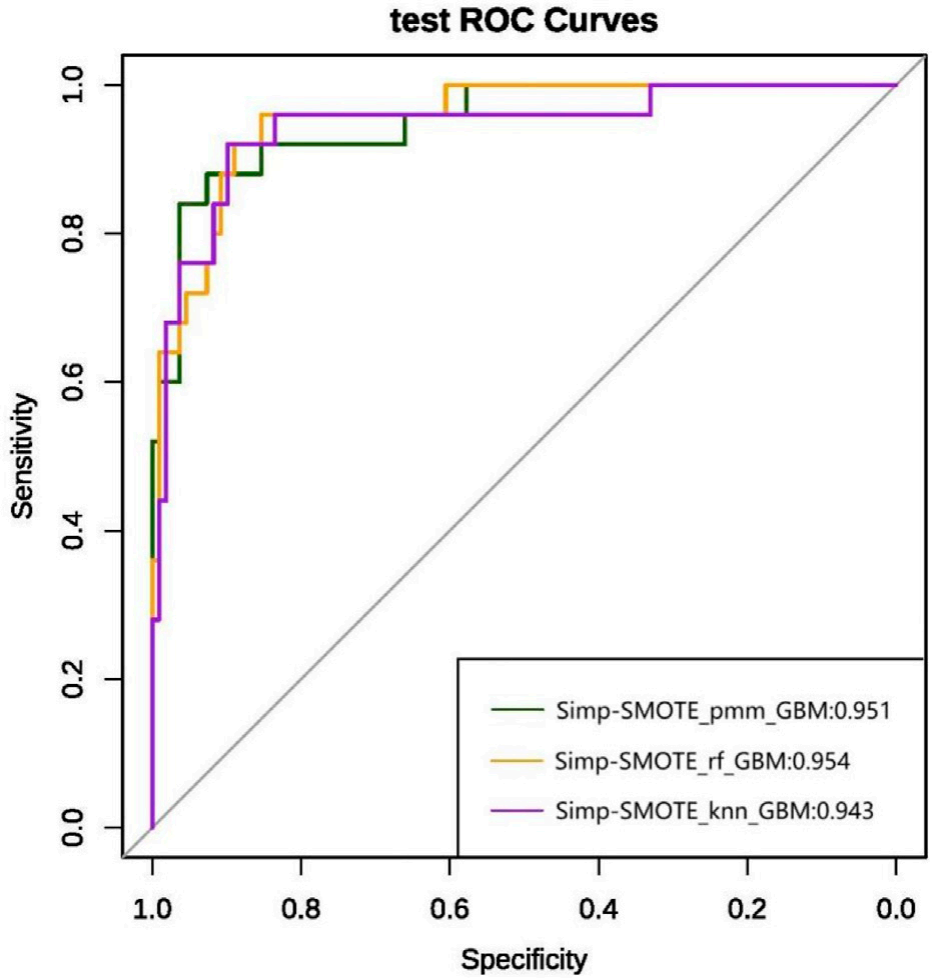
ROC curve and AUC values of the simplified model constructed based on the top nine variables in the test set.

To minimize overfitting and redundancy, simplified models were developed using only the top nine variables identified by the best-performing full model. Five simplified models were reconstructed using the same preprocessing methods and algorithms as their full counterparts. Performance metrics are presented in Table 3 and ROC curves in Figure 8. The simplified models bypassed feature selection by directly incorporating the predetermined top nine features from the comprehensive analysis. Models 1 and 2, differing only in feature selection approach but sharing k-NN imputation, SMOTE sampling, and GBM algorithm, yielded identical simplified versions (Simp-SMOTE_knn_GBM). Similarly, models four and 5 converged to the same simplified version. The Simp-SMOTE_rf_GBM model (random forest imputation + SMOTE + GBM) demonstrated superior performance across all metrics: AUC is 0.954 (95% CI: 0.932-0.976), Accuracy: 0.888, Sensitivity: 0.890, Specificity: 0.880, NPV:0.647, PPV: 0.970, and F1:0.885.

**TABLE 3:** Performance metrics results of the five simplified models reconstructed based on the first nine variables.

Model name	AUC	Accuracy	Recall	Specificity	NPV	PPV	F1
Simp-SMOTE_rf_GBM	0.954	0.888	0.890	0.880	0.647	0.970	0.885
Simp-SMOTE_rf_GBM	0.954	0.888	0.890	0.880	0.647	0.970	0.885
Simp-SMOTE_pmm_GBM	0.951	0.888	0.890	0.880	0.647	0.970	0.885
Simp-SMOTE_knn_GBM	0.943	0.903	0.899	0.920	0.676	0.980	0.909
Simp-SMOTE_knn_GBM	0.943	0.903	0.899	0.920	0.676	0.980	0.909

**FIGURE 9:**
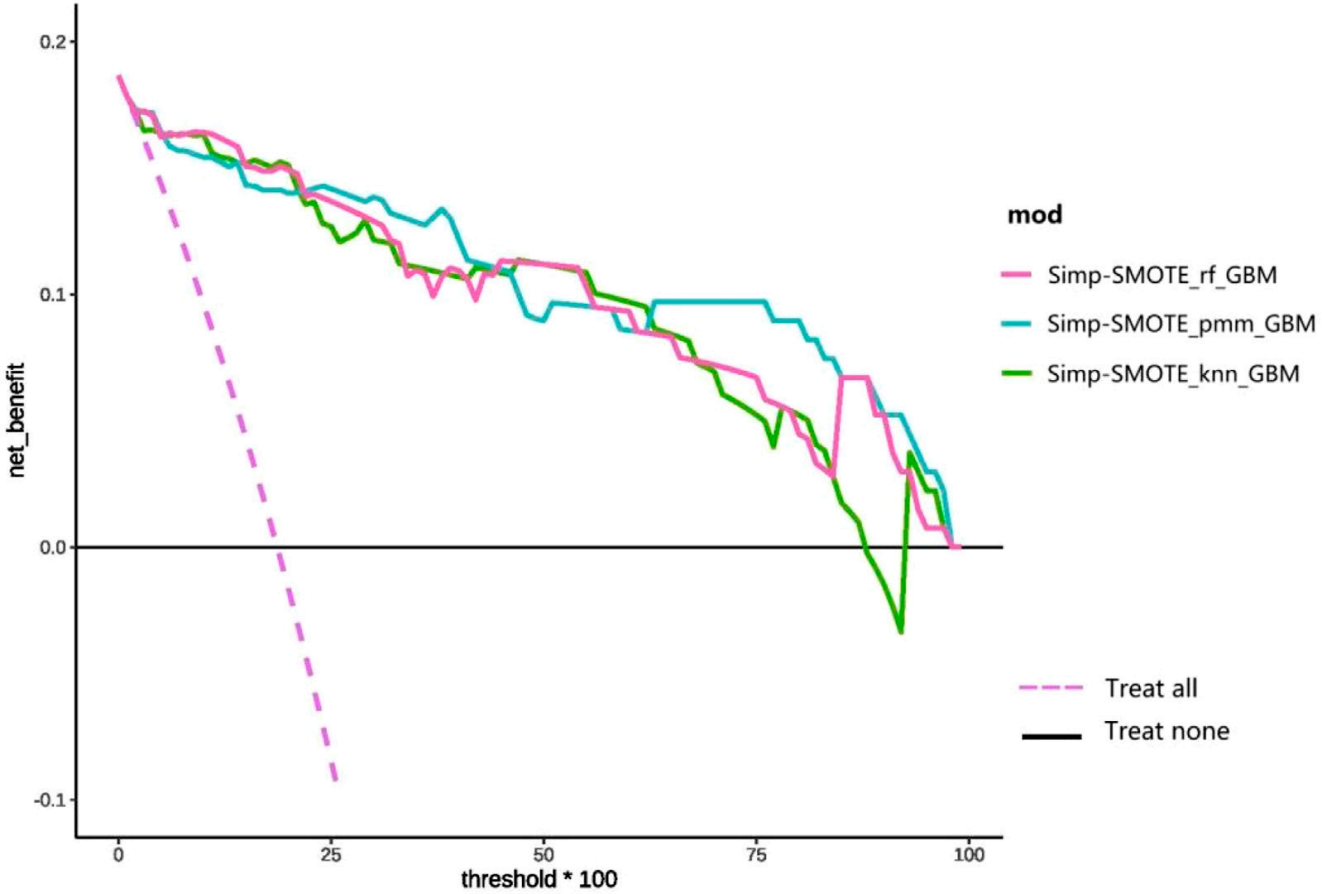
DCA curve of the simplified model reconstructed based on the first nine variables.

DCA (Figure 9) compares the clinical net benefit profiles of the simplified models across probability thresholds in the test set. The Simp-SMOTE_knn_GBM model demonstrated superior net benefit *versus* treat-all and treat-none strategies at probability thresholds of 15%–85%, suggesting optimal utility for intermediate-risk clinical decision-making. Both Simp-SMOTE_rf_GBM and Simp-SMOTE_pmm_GBM maintained clinical utility across the full threshold spectrum (0%–100%), with Simp-SMOTE_pmm_GBM showing consistently higher net benefit.

**FIGURE 10:**
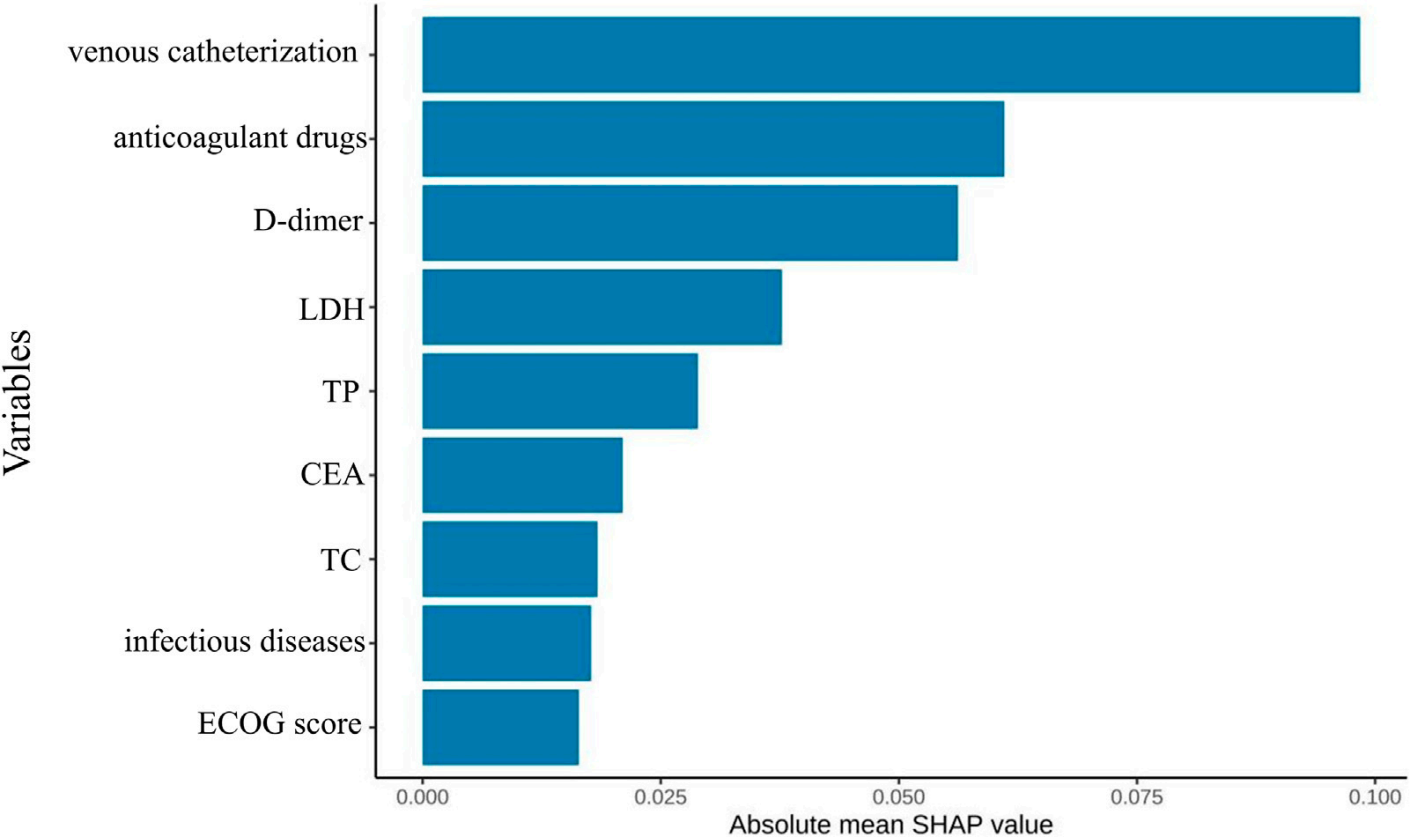
Optimal model feature importance ranking chart constructed based on the first nine feature variables.

SHAP feature importance ranking for the simplified model (Figure 10) showed central venous catheterization as the strongest predictor, followed by anticoagulant use, D-dimer, LDH, TP, CEA, cholesterol, infectious disease, and ECOG performance status.

**FIGURE 11:**
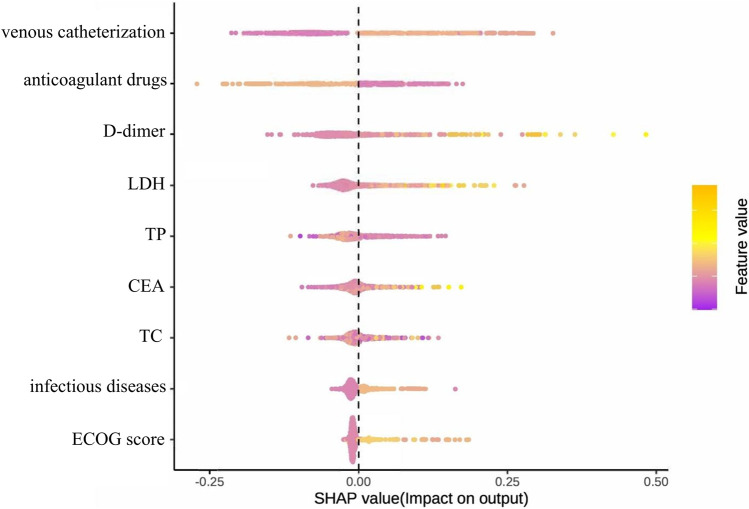
Summary diagram of the optimal model SHAP established based on the first nine feature variables.

Figure 11 presents the SHAP summary plot for the parsimonious VTE prediction model incorporating the top nine features, illustrating both feature importance and directionality of effects across the test set. The feature ranking of this simplified model differs from that of the optimal model constructed using full variable screening. Notably, active infection and TC emerged as new predictors in the simplified model, while relative importance shifted for D-dimer, therapeutic anticoagulation, and CEA. SHAP analysis revealed positive associations between VTE risk and: venous catheterization, D-dimer, LDH, CEA, and active infection. Conversely, therapeutic anticoagulation, TP, and TC demonstrated protective associations. The contribution of the ECOG score to the model output results is relatively insignificant.

### Comparison of simplified and unsimplified model performance

**FIGURE 12:**
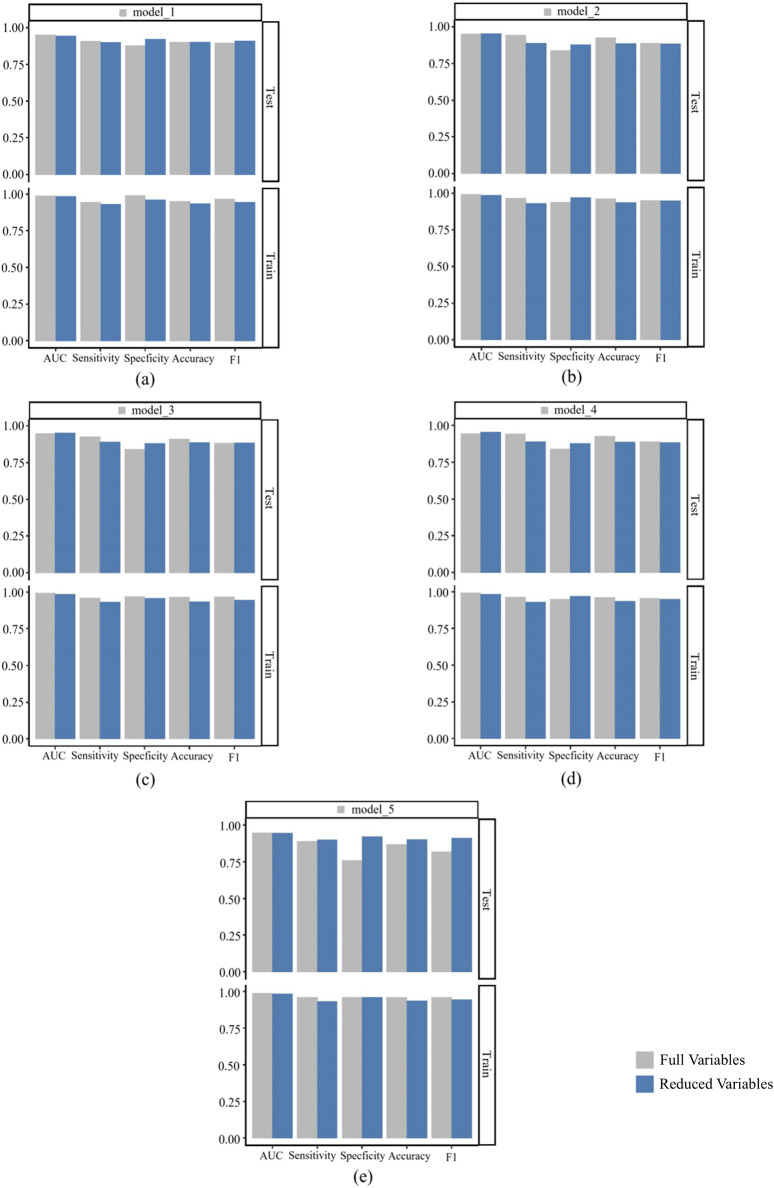
Comparison of performance indicators between the model built after full variable screening and the model built with the top nine variables. **(a)** is a comparison between model_1 and its corresponding simplified model; **(b)** is a comparison between model_2 and its corresponding simplified model; **(c)** is a comparison between model_3 and its corresponding simplified model; **(d)** is a comparison between model_4 and its corresponding simplified model; **(e)** is a comparison between model_5 and its corresponding simplified model.

We conducted a comprehensive performance comparison between the full-feature models and their simplified counterparts (using the top nine features) across five key metrics: AUC, sensitivity, specificity, accuracy, and F1-score, with detailed results shown in Figure 12. Figure 12 presents the comparative performance analysis across both training and test datasets, including 95% CI for all metrics. For the pre-simplification optimal model (a), the full-feature version demonstrated marginally superior performance compared to its simplified counterpart. Overall, the predictive performance of the simplified models differs only slightly from that of their corresponding original models, and some simplified models even outperform the original models in terms of performance metrics. These results suggest that feature reduction incurred minimal predictive penalty, while maintaining clinical utility through improved interpretability and computational efficiency.

## Discussion

Lymphoma is the most common malignant tumor of the hematopoietic system, and factors such as the high tumor burden associated with the disease itself can increase the risk of VTE. In addition, common treatment methods such as surgery, chemotherapy, and immunotherapy may also increase the risk of VTE in patients. Current VTE prevention guidelines primarily target general chronic disease populations, leaving lymphoma-specific prevention strategies inadequately addressed—a critical gap in clinical practice. There are currently comprehensive risk assessment tools for VTE in patients with lymphoma. In recent years, researchers both domestically and internationally have developed numerous VTE risk assessment tools for cancer patients. Khorana et al. conducted a large-scale retrospective cohort study involving 66,106 cancer patients with neutropenia and developed a VTE risk scoring system suitable for outpatient chemotherapy cancer patients (Streiff et al., 2021). However, two critical limitations exist: (1) exclusion of hospitalized patients potentially underestimates true VTE risk (Louzada et al., 2012), and (2) Khorana scoring studies mostly involve solid tumour patients, with only a small proportion of lymphoma patients, raising questions about its applicability in the lymphoma population. Subsequent studies by Mohren et al. involved a VTE risk prediction analysis of 2,701 patients with malignant tumours undergoing chemotherapy. However, the lymphoma subgroup accounted for only 12.1% of the total sample (Caruso et al., 2010). The Ottawa score, designed to predict 6-month VTE recurrence post-anticoagulation in cancer patients, originally dichotomized patients into low- and high-risk categories. Subsequent refinements established three risk strata (low, moderate, high) (Louzada et al., 2012; den Exter et al., 2013). However, studies by Alatri et al. (2017) have shown that the improved Ottawa score has an AUC of 0.58 (95% CI: 0.56–0.61), indicating insufficient predictive performance. The accuracy and discriminative ability of the score in predicting VTE recurrence are generally poor, with low sensitivity, specificity, and positive predictive value. It is unable to accurately predict VTE recurrence in patients with cancer-related thrombosis, which could lead to biased clinical decision-making.

Due to natural differences in ethnicity and genetics, certain models are not applicable in Chinese cohorts. However, the universality of pan-cancer models has been called into question in the context of lymphoma. Validation studies demonstrate superior discrimination for lymphoma-specific models (e.g., TiC-LYMPHO: C-statistic 0.783, 95% CI: 0.752-0.814) *versus* pan-cancer tools in lymphoma populations (Bastos-Oreiro et al., 2021). Lymphoma-specific VTE risk factors (e.g., LDH, β2-microglobulin) differ substantially from solid tumors (Antic et al., 2016; Lim et al., 2016; Dharmavaram et al., 2020), explaining the poor performance of pan-cancer models. Key lymphoma biomarkers, including LDH and β2-microglobulin, demonstrate VTE associations (Yıldız et al., 2020), yet remain absent from general cancer models. Suboptimal risk stratification may cause both under-anticoagulation and over-treatment, adversely impacting clinical outcomes and healthcare costs. This study uses machine learning algorithms to analyse real-world medical data and incorporate multiple types of variables in order to develop a VTE risk prediction model for lymphoma patients. The model aims to provide clinicians with an intelligent decision-support tool that optimises strategies for preventing thrombosis and reduces the incidence of bleeding events.

Through systematic evaluation of 27 preprocessing pipelines and nine machine learning algorithms, we developed and validated 243 distinct prediction models. Although the optimal full-variable model (KNN interpolation + SMOTE + elastic network + gradient boosting machine) achieved an AUC of 0.953, there is a risk of feature redundancy. Therefore, based on feature importance, the top nine key variables were selected to rebuild a simplified model, ultimately obtaining the Simp-SMOTE_rf_GBM model (AUC = 0.954), which demonstrated superior predictive performance and practicality compared to the full-variable model. The simplified model showed robust external validity (calibration slope 0.98) while preserving accuracy (0.888) and sensitivity (0.890), making it particularly suitable for clinical implementation. Among the key features, venous catheterization, D-dimer, and LDH were positively correlated with VTE risk, while anticoagulant drugs and TP were negatively correlated. Feature importance hierarchy shifted in the simplified model, with TC and active infection replacing β2-microglobulin and hematopoietic growth factors. SHAP analysis further validated the contribution direction of these features. Key predictors such as venous catheterization, anticoagulant drugs, TC, and infectious diseases have special significance in clinical practice.

Venous catheterization is primarily used for patients requiring long-term intravenous infusion therapy, such as chemotherapy or intravenous nutrition. Currently, the most commonly used central venous catheter access routes in clinical practice include central venous catheters (CVCs) inserted via the internal jugular vein, subclavian vein, or femoral vein, and peripherally inserted central venous catheters (PICCs). Research data shows that venous catheterization may cause a 40%–80% decrease in venous blood flow rate. When combined with cancer-associated hypercoagulability, these hemodynamic changes synergistically increase thrombosis risk, potentially leading to life-threatening PE (Guan et al., 2018; Yang et al., 2020). Currently, multiple evidence-based medical studies have confirmed that venous catheterization is an independent risk factor for VTE in lymphoma patients (Park et al., 2012; Guan et al., 2018; Kirkizlar et al., 2020; Wan, 2024). Lymphoma patients receiving PICCs demonstrate a 5.25-fold increased VTE risk (95% CI 3.8-7.1) compared to non-catheterized patients (Zhang, 2025). Park et al. (2012) reported CVC-associated thrombosis risk elevation (OR 2.04, 95% CI 1.02-4.08, p = 0.042) in a prospective cohort of 452 lymphoma patients. A Chinese study, however, showed that the likelihood of VTE in lymphoma patients using CVC was 6.63 times higher than in those not using CVC (OR = 6.63, 95% CI: 2.24–19.57, p = 0.001) (Y et al., 2021).

According to clinical practice guidelines, the prophylactic use of anticoagulants can effectively reduce the risk of VTE and is an important protective factor (Streiff et al., 2021). Our findings corroborate guideline recommendations, showing significantly elevated VTE risk in patients without prophylaxis. Low molecular weight heparin (LMWH) drugs such as enoxaparin and nadroparin are currently the drugs of choice for prophylactic anticoagulant therapy. LMWHs exert their antithrombotic effect through selective inhibition of factor Xa and factor IIa, effectively interrupting the coagulation cascade. In recent years, direct oral anticoagulants (DOACs), such as rivaroxaban and apixaban, have become more widely used in clinical practice. These drugs offer advantages such as convenient administration and the elimination of the need for frequent monitoring of coagulation parameters. Studies have shown that they are as effective as LMWH at preventing blood clots in patients with malignant tumours (Agnelli et al., 2020). However, anticoagulation carries inherent bleeding risks, particularly in patients with thrombocytopenia, a history of gastrointestinal ulcers, or recent surgical procedures. Therefore, before initiating anticoagulant prophylaxis, it is essential to conduct a comprehensive assessment of the patient’s bleeding and thrombosis risks, and to develop a personalised treatment plan. Furthermore, the development of safer, easier-to-use anticoagulant drugs is necessary in order to provide lymphoma patients with an optimised thrombosis prevention regimen. Ying L et al. (2024) found that prophylactic anticoagulant use is a significant protective factor against PICC-related thrombosis in cancer patients. This factor served as the primary node in their decision tree model, underscoring its significant impact on thrombus formation. Furthermore, Boraks et al. (Boraks et al., 1998) demonstrated that prophylactic low-dose warfarin effectively reduces the incidence of catheter-related thrombosis. However, Heaton et al. (2002) found that a low-dose warfarin regimen (1 mg) did not significantly inhibit catheter-related thrombotic events in cancer patients. Therefore, the efficacy of prophylactic anticoagulation in reducing venous thrombosis incidence requires further validation and investigation in larger, prospective studies.

Lymphoma patients frequently exhibit compromised immune function and substantially elevated infection risk attributable to both the malignancy itself and treatment-related factors, including chemotherapy and immunosuppressive agents. Such infections further exacerbate VTE incidence, adversely impacting tumor prognosis. Multiple studies establish concomitant infections as significant risk factors for DVT in cancer patients (Chen et al., 2020; Wang et al., 2023). Patients with concomitant infections exhibit 2- to 3-fold higher VTE incidence compared to infection-free patients. This correlation is particularly pronounced within the initial 6 months post-diagnosis (Nakano et al., 2018). The prothrombotic effects of infection are multifactorial, encompassing oxidative stress, systemic inflammation, coagulation activation, and endothelial injury. Inflammatory responses—driven in part by neutrophil-derived cytokines such as interleukin-6 and tumor necrosis factor–α—induce tissue factor expression in circulating monocytes and promote release of tissue factor from monocytes and platelets, thereby activating the extrinsic coagulation pathway, fostering fibrin formation, and suppressing fibrinolysis to create a hypercoagulable state (Lim et al., 2016). In parallel, infection triggers innate immune pathways involving neutrophils and the formation of neutrophil extracellular traps (NETs), which help immobilize pathogens within the vasculature but also amplify thrombin generation and thrombosis (Longstaff et al., 2013). Together, these processes lead to endothelial injury, platelet activation and aggregation, increased procoagulant protein activity, and attenuation of anticoagulant mechanisms, culminating in thrombus formation (Beristain-Covarrubias et al., 2019). Overall, infection is both a potent precipitant of VTE in lymphoma and a key determinant of adverse prognosis.

CEA is a glycoprotein of the immunoglobulin superfamily that participates in cell adhesion, inflammatory signaling, and tumor progression (Kankanala et al., 2025). In a study of patients with lung cancer, multivariable analyses demonstrated a linear positive association between CEA concentration and pulmonary embolism, suggesting that elevated CEA may help identify individuals at increased risk of PE (Zhang et al., 2014). As a nonspecific tumor marker, CEA reflects tumor burden and growth kinetics and is widely used for diagnosis and prognostication. However, evidence linking CEA to VTE risk remains limited, and its clinical significance is not fully established. Large, prospective cohort studies are needed to clarify the mechanistic relationship between CEA and thrombogenesis.

Age (Chen et al., 2022),inherited predisposition (Sánchez Prieto et al., 2024),and reduced mobility (Saito et al., 2021) are established risk factors for VTE in patients with lymphoma. In one study, individuals with lymphoma or multiple myeloma who carried factor V Leiden and SERPINA10 variants had higher VTE incidence; other data indicate that the coexistence of cancer and factor V Leiden variants synergistically increases VTE risk (Gran et al., 2016). In our feature-selection pipeline, however, these variables were not retained in the final predictive model, likely due to sample characteristics, collinearity, or methodological constraints. Their exclusion does not diminish their clinical relevance in lymphoma, and their potential contributions warrant attention. To define the magnitude and independence of these associations, adequately powered, prospective, multicenter studies are needed.

This study has multifaceted clinical applicability. Firstly, the model helps to identify high-risk patients, supporting the personalisation of decisions regarding prophylactic anticoagulation. Secondly, it incorporates dynamic variables, such as changes in D-dimer levels, which enable ongoing risk assessment during treatment. Furthermore, the risk factors elucidated by the model could be used to refine VTE prevention strategies. For example, more aggressive prophylactic anticoagulation could be considered for patients requiring mandatory venous catheterisation. Finally, the simplified version of the model, which includes only nine readily available clinical variables, significantly enhances its feasibility for implementation in settings with limited resources.

## Limitations

This study has limitations. Firstly, as this was a retrospective analysis, several clinically relevant variables, e.g., genetic risk scores, immunophenotypic scores (IPS), Throly scores and Khorana scores, were either unavailable or severely lacking because some assessments are not routinely performed in patients with lymphoma in the absence of clear clinical indications. Consequently, they were excluded from modelling and infrequently used medications were aggregated into composite categories. Secondly, VTE ascertainment from the electronic medical record may have missed events (e.g., non-specific symptoms, lack of screening or diagnoses made at outside institutions), which would lead to an underestimation of incidence. The small number of VTE cases also produced class imbalance, which may persist despite Synthetic Minority Over-sampling Technique (SMOTE) correction and could affect model performance. Thirdly, this was a single-centre study with a limited sample size and few VTE events. Future work should employ larger, prospective, multicentre cohorts to validate, refine and generalise the model.

## Conclusion

This study developed a VTE risk prediction model specifically for lymphoma patients. With an AUC of 0.953, the optimized model exhibited outstanding discriminative capacity for lymphoma-associated VTE risk, providing an evidence-based framework to guide optimal timing of anticoagulation initiation and clinical strategy formulation. This model enables theoretically grounded and clinically actionable VTE risk stratification in lymphoma populations, advancing precision medicine approaches to ultimately enhance clinical outcomes.

Multicenter prospective validation warrants prioritization to establish model generalizability and robustness across heterogeneous healthcare environments. Concurrent integration of multi-omics data with emerging biomarkers—including circulating tumor DNA (ctDNA), microvesicles, and novel coagulation parameters (thrombin-antithrombin complex [TAT], plasmin-α_2_-plasmin inhibitor complex [PIC], thrombomodulin [TM], tissue plasminogen activator inhibitor complex [tPAI·C])—may substantially improve prognostic precision. Intervention trials should evaluate risk-stratified anticoagulation protocols via randomized controlled designs, validating their efficacy in optimizing hard clinical endpoints. Mechanistic studies must elucidate lymphoma-specific prothrombotic pathways, focusing on coagulation biomarkers (e.g., TAT, PIC) within tumor-associated thrombosis to inform targeted therapeutic development. These integrated approaches will accelerate the evolution of precision medicine in lymphoma-associated VTE management.

## Data Availability

The data for this study were obtained from Sichuan Provincial People’s Hospital and are not publicly available. However, they can be obtained from the corresponding author upon reasonable request.
