# Supplementary material for: A study on a real-world data-based VTE risk prediction model for lymphoma patients
Source: Front Pharmacol. 2025 Oct 14;16:1691271. doi: 10.3389/fphar.2025.1691271 (PMC12558764; doi:10.3389/fphar.2025.1691271)
Supplement: Supplementary file 1 [file Supplementaryfile1.docx]

Supplementary Material

**Table S1.** **Data cleaning methods and algorithm ID assignment.**

| **Method** | **Name** | **Parameters** | **ID** |
| --- | --- | --- | --- |
| **Imputation Method** | K-Nearest Neighbors Imputation | KNN | 0 |
|  | Random Forest Imputation | RF | 1 |
|  | Stochastic Regression Imputation | PMM | 2 |
| **Sampling Method** | Random Oversampling | ROS | 0 |
|  | SMOTE Oversampling | SOM | 1 |
|  | Borderline SMOTE Oversampling | BSOM | 2 |
| **Feature Selection** | Lasso Feature Selection | LA | 0 |
|  | Ridge Feature Selection | RD | 1 |
|  | ElasticNet Feature Selection | EN | 2 |
| **Algorithm** | Logistic Regression | LR | 0 |
|  | Decision Tree | DT | 1 |
|  | Random Forest | RF | 2 |
|  | Support Vector Machine | SVM | 3 |
|  | Naive Bayes | NB | 4 |
|  | K-Nearest Neighbors | KNN | 5 |
|  | Gradient Boosting Machine | GBM | 6 |
|  | Extreme Gradient Boosting | XGB | 7 |
|  | Adaptive Boosting | AdaBoost | 8 |

**Table S2. Evaluation Indicator Calculation Formula**

| **Name** | **Calculation formula** |
| --- | --- |
| $\mathrm{Accuracy}$ | $\frac{\mathrm{TP}+\mathrm{TN}}{\mathrm{TP}+\mathrm{TN}+\mathrm{FP}+\mathrm{FN}}$ |
| $\mathrm{Recall}$ | $\frac{\mathrm{TP}}{TP+FN}$ |
| $\mathrm{Specificity}$ | $\frac{\mathrm{TN}}{\mathrm{TN}+\mathrm{FP}}$ |
| $\mathrm{NPV}$ | $\frac{\mathrm{TN}}{\mathrm{TN}+\mathrm{FN}}$ |
| $\mathrm{PPV}$ | $\frac{\mathrm{TP}}{\mathrm{TP}+\mathrm{FP}}$ |
| $F1$ | $\frac{2Precision\times Recall}{Precision+Recall}$ |

NOTE: True Positive, TP; False Positive, FP; True Negative, TN; False Negative, FN

**Table S3. Characteristics of patients with positive lymphoma**

| **Patient Characteristics** | **Cases (n)** | **Proportion (%)** |
| --- | --- | --- |
| **Histological Subtype** |  |  |
| Aggressive Lymphoma | 51 | 83.6 |
| Indolent Lymphoma | 9 | 14.8 |
| Not Specified | 1 | 1.6 |
| **Tumor Stage** |  |  |
| Stage I-II | 43 | 70.5 |
| Stage III-IV | 11 | 18.0 |
| Not Specified | 7 | 11.5 |
| **Time to VTE Occurrence** |  |  |
| ≤30 days | 28 | 45.9 |
| 31-60 days | 17 | 27.9 |
| 61-180 days | 16 | 26.2 |
| **VTE Location Distribution** |  |  |
| **Single Site** |  |  |
| Cranial/Neck Veins | 6 | 9.8 |
| Upper Extremity DVT | 11 | 18.0 |
| Portal Vein Thrombosis | 2 | 3.3 |
| Lower Extremity DVT | 30 | 49.2 |
| PE | 5 | 8.2 |
| **Multiple Sites** |  |  |
| Multisite Venous Thrombosis | 7 | 11.5 |
| Cranial/Neck + UE DVT | 3 | 4.9 |
| Cranial/Neck + LE DVT | 2 | 3.3 |
| PE + LE DVT | 1 | 1.6 |
| Cranial/Neck+UE+LE DVT | 1 | 1.6 |

**Table S4. Results of Univariate Analysis for Patient Characteristics**

| **Characteristic** | **Non-VTE Group (N=544)** | **VTE Group (N=61)** | ***P*-value** |
| --- | --- | --- | --- |
|  |  |  |  |
| Age | 54.92±14.53 | 59.72±12.76 | 0.003 |
| Height | 162.51±9.94 | 162.23±7.28 | 0.376 |
| Weight | 60.09±10.18 | 60.55±11.46 | 0.561 |
| BMI | 22.72±3.20 | 22.93±3.64 | 0.882 |
| Sex |  |  |  |
| Female | 254(46.69%) | 23(37.70%) | 0.182 |
| Male | 290(53.31%) | 38(62.30%) |  |
| Smoking History |  |  |  |
| Yes | 149(27.39%) | 18(29.51%) | 0.725 |
| No | 395(72.61%) | 43(70.49%) |  |
| Chronic Alcohol Use |  |  |  |
| Yes | 75(13.79%) | 10(16.39%) | 0.579 |
| No | 469(86.21%) | 51(83.61%) |  |
| ECOG score |  |  |  |
| <2 | 244(83.85%) | 27(67.50%) | 0.012 |
| ≥2 | 47(16.15%) | 13(32.50%) |  |
| Hypertension |  |  |  |
| Present | 99(18.20%) | 16(26.23%) | 0.130 |
| Absent | 445(81.80%) | 459(73.77%) |  |
| Diabetes Mellitus |  |  |  |
| Present | 60(11.03%) | 12(19.67%) | 0.048 |
| Absent | 484(88.97%) | 49(80.33%) |  |
| Infectious Diseases |  |  |  |
| Present | 154(28.31%) | 31(50.82%) | ＜0.001 |
| Absent | 390(71.69%) | 30(49.18%) |  |
| Hepatic Disorders |  |  |  |
| Present | 156(28.68%) | 25(40.98%) | 0.047 |
| Absent | 388(71.32%) | 36(59.02%) |  |
| Electrolyte Imbalance |  |  |  |
| Present | 95(17.46%) | 18(29.51%) | 0.022 |
| Absent | 449(82.54%) | 43(70.49%) |  |
| Pulmonary Diseases |  |  |  |
| Present | 70(12.87%) | 11(18.03%) | 0.261 |
| Absent | 474(87.13%) | 50(81.97%) |  |
| Transfusion History |  |  |  |
| Yes | 57(10.48%) | 12(19.67%) | 0.032 |
| No | 487(89.52%) | 49(80.33%) |  |
| Histological Subtype |  |  |  |
| Hodgkin Lymphoma | 25(4.68%) | 0(0.00%) | 0.008 |
| Aggressive Lymphoma | 359(67.23%) | 51(85.00%) |  |
| Indolent Lymphoma | 150(28.09%) | 9(15.00%) |  |
| Ann Arbor Stage |  |  |  |
| Stage I-II | 186(36.47%) | 11(20.37%) | 0.018 |
| Stage III-IV | 324(63.53%) | 43(79.63%) |  |
| Relapsed/Refractory Lymphoma |  |  |  |
| Yes | 20(3.68%) | 4(6.56%) | 0.455 |
| No | 524(96.32%) | 57(93.44%) |  |
| Extranodal Involvement |  |  |  |
| Present | 368(71.32%) | 45(83.33%) | 0.060 |
| Absent | 148(28.68%) | 9(16.67%) |  |
| Mediastinal Involvement |  |  |  |
| Present | 157(30.37%) | 20(35.71%) | 0.411 |
| Absent | 360(69.63%) | 36(64.29%) |  |
| Bone Marrow Involvement |  |  |  |
| Present | 147(29.28%) | 19(38.78%) | 0.167 |
| Absent | 355(70.72%) | 30(61.22%) |  |
| Central Nervous System (CNS) Involvement |  |  |  |
| Present | 16(2.95%) | 7(11.48%) | 0.003 |
| Absent | 527(97.05%) | 54(88.52%) |  |
| Splenic Involvement |  |  |  |
| Present | 125(23.54%) | 15(26.32%) | 0.640 |
| Absent | 406(76.46%) | 42(73.68%) |  |
| B Symptoms |  |  |  |
| Present | 170(32.38%) | 20(33.90%) | 0.998 |
| Absent | 355(67.62%) | 39(66.10%) |  |
| Bulky Disease (>10 cm) |  |  |  |
| Present | 80(15.01%) | 9(15.00%) | 0.814 |
| Absent | 453(84.99%) | 51(85.00%) |  |
| Platinum-based Agents |  |  |  |
| Received | 67(12.32%) | 8(13.11%) | 0.858 |
| Not Received | 477(87.68%) | 53(86.89%) |  |
| Anthracyclines |  |  |  |
| Received | 364(66.91%) | 44(72.13%) | 0.409 |
| Not Received | 180(33.09%) | 17(27.87%) |  |
| Rituximab |  |  |  |
| Received | 386(70.96%) | 43(70.49%) | 0.940 |
| Not Received | 158(29.04%) | 18(29.51%) |  |
| ESAs/G-CSF |  |  |  |
| Received | 255(46.88%) | 37(60.66%) | 0.004 |
| Not Received | 319(58.64%) | 24(39.34%) |  |
| Methotrexate |  |  |  |
| Received | 103(18.93%) | 15(24.59%) | 0.29 |
| Not Received | 441(81.07%) | 46(75.41%) |  |
| Cyclophosphamide |  |  |  |
| Received | 367(67.46%) | 45(73.77%) | 0.316 |
| Not Received | 177(32.54%) | 16(26.23%) |  |
| Anticoagulants |  |  |  |
| Received | 207(38.05%) | 14(22.95%) | 0.02 |
| Not Received | 337(61.95%) | 47(77.05%) |  |
| Venous Catheterization |  |  |  |
| Performed | 196(36.03%) | 39(63.93%) | ＜0.001 |
| Not Performed | 348(63.97%) | 22(36.07%) |  |
| General Anesthesia Surgery |  |  |  |
| Performed | 112(20.59%) | 17(27.87%) | 0.191 |
| Not Performed | 432(79.41%) | 44(72.13%) |  |
| D-dimer | 1.45±2.85 | 4.81±6.23 | ＜0.001 |
| White Blood Cell Count | 8.42±12.66 | 12.28±20.08 | 0.222 |
| Platelet Count | 195.03±95.04 | 170.93±99.39 | 0.275 |
| Hemoglobin Concentration | 326.14±21.10 | 321.26±14.87 | 0.289 |
| Fibrinogen | 3.60±1.37 | 3.74±1.98 | 0.553 |
| Neutrophil Count | 4.55±2.74 | 5.22±4.10 | 0.147 |
| Monocyte Count | 0.50±0.41 | 0.61±0.63 | 0.064 |
| Red Blood Cell Count | 4.08±0.80 | 3.68±1.05 | 0.008 |
| Hematocrit | 36.14±8.12 | 32.32±8.49 | 0.005 |
| Alanine Aminotransferase | 29.79±47.66 | 31.47±30.27 | 0.499 |
| Albumin | 38.64±5.96 | 35.65±6.01 | ＜0.001 |
| Total Protein | 68.00±9.29 | 63.11±8.34 | ＜0.001 |
| Activated Partial Thromboplastin Time | 27.29±3.77 | 27.15±3.72 | 0.392 |
| Activated Partial Thromboplastin Ratio | 0.99±0.17 | 0.98±0.11 | 0.707 |
| Prothrombin Time | 11.81±7.03 | 11.91±1.76 | 0.062 |
| Thrombin Time | 17.86±13.74 | 16.96±1.28 | 0.293 |
| International Normalized Ratio | 1.04±0.14 | 1.07±0.16 | 0.092 |
| Fibrin Degradation Products | 4.92±9.17 | 10.91±13.71 | ＜0.001 |
| High-sensitivity C-Reactive Protein | 20.57±35.60 | 39.85±47.13 | ＜0.001 |
| Carcinoembryonic Antigen | 2.02±1.24 | 2.81±2.94 | 0.335 |
| Lactate Dehydrogenase | 330.51±325.30 | 612.79±576.80 | ＜0.001 |
| Creatinine | 67.95±35.47 | 77.67±47.85 | 0.118 |
| High-sensitivity Troponin I | 5.20±27.06 | 22.69±95.33 | 0.385 |
| Total Cholesterol | 3.61±1.18 | 3.84±1.12 | 0.237 |
| Serum Calcium | 2.24±0.19 | 2.18±0.19 | 0.001 |
| Homocysteine | 13.47±6.48 | 15.60±6.83 | 0.319 |
| Antithrombin III | 87.17±14.93 | 82.68±12.18 | 0.086 |
| β-2 Microglobulin | 3.48±2.68 | 4.90±3.03 | 0.007 |

**Table S5. Comparison of the Performance of 243 Risk Prediction Models Constructed Using All Variables**

| **Imputation Method** | **Sampling Method** | **Feature Selection** | **Algorithm** | **AUC** | **Accuracy** | **Recall** | **Specificity** | **NPV** | **PPV** | **F1** |
| --- | --- | --- | --- | --- | --- | --- | --- | --- | --- | --- |
| 0 | 0 | 0 | 0 | 0.865 | 0.773 | 0.784 | 0.714 | 0.935 | 0.385 | 0.747 |
| 0 | 0 | 0 | 1 | 0.727 | 0.833 | 0.901 | 0.476 | 0.901 | 0.476 | 0.623 |
| 0 | 0 | 0 | 2 | 0.882 | 0.879 | 0.901 | 0.762 | 0.952 | 0.593 | 0.826 |
| 0 | 0 | 0 | 3 | 0.912 | 0.864 | 0.865 | 0.857 | 0.970 | 0.545 | 0.861 |
| 0 | 0 | 0 | 4 | 0.694 | 0.811 | 0.865 | 0.524 | 0.906 | 0.423 | 0.652 |
| 0 | 0 | 0 | 5 | 0.617 | 0.811 | 0.901 | 0.333 | 0.877 | 0.389 | 0.487 |
| 0 | 0 | 0 | 6 | 0.915 | 0.924 | 0.955 | 0.762 | 0.955 | 0.762 | 0.848 |
| 0 | 0 | 0 | 7 | 0.841 | 0.894 | 0.928 | 0.714 | 0.945 | 0.652 | 0.807 |
| 0 | 0 | 0 | 8 | 0.828 | 0.848 | 0.892 | 0.619 | 0.925 | 0.520 | 0.731 |
| 0 | 0 | 1 | 0 | 0.671 | 0.773 | 0.820 | 0.524 | 0.901 | 0.355 | 0.639 |
| 0 | 0 | 1 | 1 | 0.701 | 0.818 | 0.883 | 0.476 | 0.899 | 0.435 | 0.619 |
| 0 | 0 | 1 | 2 | 0.902 | 0.939 | 0.964 | 0.810 | 0.964 | 0.810 | 0.880 |
| 0 | 0 | 1 | 3 | 0.855 | 0.758 | 0.721 | 0.952 | 0.988 | 0.392 | 0.821 |
| 0 | 0 | 1 | 4 | 0.565 | 0.788 | 0.892 | 0.238 | 0.861 | 0.294 | 0.376 |
| 0 | 0 | 1 | 5 | 0.769 | 0.871 | 0.919 | 0.619 | 0.927 | 0.591 | 0.740 |
| 0 | 0 | 1 | 6 | 0.915 | 0.917 | 0.955 | 0.714 | 0.946 | 0.750 | 0.817 |
| 0 | 0 | 1 | 7 | 0.766 | 0.811 | 0.865 | 0.524 | 0.906 | 0.423 | 0.652 |
| 0 | 0 | 1 | 8 | 0.837 | 0.826 | 0.856 | 0.667 | 0.931 | 0.467 | 0.750 |
| 0 | 0 | 2 | 0 | 0.865 | 0.818 | 0.838 | 0.714 | 0.939 | 0.455 | 0.771 |
| 0 | 0 | 2 | 1 | 0.734 | 0.871 | 0.946 | 0.476 | 0.905 | 0.625 | 0.633 |
| 0 | 0 | 2 | 2 | 0.871 | 0.939 | 0.973 | 0.762 | 0.956 | 0.842 | 0.855 |
| 0 | 0 | 2 | 3 | 0.918 | 0.879 | 0.883 | 0.857 | 0.970 | 0.581 | 0.870 |
| 0 | 0 | 2 | 4 | 0.723 | 0.826 | 0.874 | 0.571 | 0.915 | 0.462 | 0.691 |
| 0 | 0 | 2 | 5 | 0.627 | 0.795 | 0.874 | 0.381 | 0.882 | 0.364 | 0.531 |
| 0 | 0 | 2 | 6 | 0.934 | 0.864 | 0.883 | 0.762 | 0.951 | 0.552 | 0.818 |
| 0 | 0 | 2 | 7 | 0.841 | 0.864 | 0.883 | 0.762 | 0.951 | 0.552 | 0.818 |
| 0 | 0 | 2 | 8 | 0.828 | 0.848 | 0.892 | 0.619 | 0.925 | 0.520 | 0.731 |
| 0 | 1 | 0 | 0 | 0.829 | 0.788 | 0.793 | 0.762 | 0.946 | 0.410 | 0.777 |
| 0 | 1 | 0 | 1 | 0.779 | 0.833 | 0.874 | 0.619 | 0.924 | 0.481 | 0.725 |
| 0 | 1 | 0 | 2 | 0.920 | 0.947 | 0.982 | 0.762 | 0.956 | 0.889 | 0.858 |
| 0 | 1 | 0 | 3 | 0.865 | 0.848 | 0.865 | 0.762 | 0.950 | 0.516 | 0.810 |
| 0 | 1 | 0 | 4 | 0.708 | 0.833 | 0.892 | 0.524 | 0.908 | 0.478 | 0.660 |
| 0 | 1 | 0 | 5 | 0.604 | 0.788 | 0.874 | 0.333 | 0.874 | 0.333 | 0.483 |
| 0 | 1 | 0 | 6 | 0.925 | 0.902 | 0.928 | 0.762 | 0.954 | 0.667 | 0.837 |
| 0 | 1 | 0 | 7 | 0.881 | 0.848 | 0.856 | 0.810 | 0.960 | 0.515 | 0.832 |
| 0 | 1 | 0 | 8 | 0.875 | 0.902 | 0.964 | 0.571 | 0.922 | 0.750 | 0.718 |
| 0 | 1 | 1 | 0 | 0.658 | 0.795 | 0.865 | 0.429 | 0.889 | 0.375 | 0.573 |
| 0 | 1 | 1 | 1 | 0.843 | 0.894 | 0.928 | 0.714 | 0.945 | 0.652 | 0.807 |
| 0 | 1 | 1 | 2 | 0.898 | 0.947 | 0.973 | 0.810 | 0.964 | 0.850 | 0.884 |
| 0 | 1 | 1 | 3 | 0.860 | 0.856 | 0.910 | 0.571 | 0.918 | 0.545 | 0.702 |
| 0 | 1 | 1 | 4 | 0.613 | 0.803 | 0.892 | 0.333 | 0.876 | 0.368 | 0.485 |
| 0 | 1 | 1 | 5 | 0.736 | 0.848 | 0.901 | 0.571 | 0.917 | 0.522 | 0.699 |
| 0 | 1 | 1 | 6 | 0.903 | 0.909 | 0.955 | 0.667 | 0.938 | 0.737 | 0.785 |
| 0 | 1 | 1 | 7 | 0.805 | 0.886 | 0.964 | 0.476 | 0.907 | 0.714 | 0.637 |
| 0 | 1 | 1 | 8 | 0.844 | 0.902 | 0.964 | 0.571 | 0.922 | 0.750 | 0.718 |
| 0 | 1 | 2 | 0 | 0.801 | 0.795 | 0.820 | 0.667 | 0.929 | 0.412 | 0.735 |
| 0 | 1 | 2 | 1 | 0.779 | 0.833 | 0.874 | 0.619 | 0.924 | 0.481 | 0.725 |
| 0 | 1 | 2 | 2 | 0.925 | 0.924 | 0.955 | 0.762 | 0.955 | 0.762 | 0.848 |
| 0 | 1 | 2 | 3 | 0.861 | 0.795 | 0.793 | 0.810 | 0.957 | 0.425 | 0.801 |
| 0 | 1 | 2 | 4 | 0.714 | 0.811 | 0.856 | 0.571 | 0.913 | 0.429 | 0.685 |
| 0 | 1 | 2 | 5 | 0.604 | 0.788 | 0.874 | 0.333 | 0.874 | 0.333 | 0.483 |
| 0 | 1 | 2 | 6 | 0.917 | 0.902 | 0.928 | 0.762 | 0.954 | 0.667 | 0.837 |
| 0 | 1 | 2 | 7 | 0.869 | 0.841 | 0.874 | 0.667 | 0.933 | 0.500 | 0.756 |
| 0 | 1 | 2 | 8 | 0.881 | 0.871 | 0.919 | 0.619 | 0.927 | 0.591 | 0.740 |
| 0 | 2 | 0 | 0 | 0.866 | 0.811 | 0.811 | 0.810 | 0.957 | 0.447 | 0.810 |
| 0 | 2 | 0 | 1 | 0.698 | 0.818 | 0.883 | 0.476 | 0.899 | 0.435 | 0.619 |
| 0 | 2 | 0 | 2 | 0.893 | 0.902 | 0.928 | 0.762 | 0.954 | 0.667 | 0.837 |
| 0 | 2 | 0 | 3 | 0.914 | 0.955 | 0.991 | 0.762 | 0.957 | 0.941 | 0.861 |
| 0 | 2 | 0 | 4 | 0.680 | 0.818 | 0.883 | 0.476 | 0.899 | 0.435 | 0.619 |
| 0 | 2 | 0 | 5 | 0.636 | 0.811 | 0.892 | 0.381 | 0.884 | 0.400 | 0.534 |
| 0 | 2 | 0 | 6 | 0.908 | 0.909 | 0.937 | 0.762 | 0.954 | 0.696 | 0.840 |
| 0 | 2 | 0 | 7 | 0.814 | 0.879 | 0.937 | 0.571 | 0.920 | 0.632 | 0.710 |
| 0 | 2 | 0 | 8 | 0.883 | 0.909 | 0.919 | 0.857 | 0.971 | 0.667 | 0.887 |
| 0 | 2 | 1 | 0 | 0.675 | 0.697 | 0.721 | 0.571 | 0.899 | 0.279 | 0.637 |
| 0 | 2 | 1 | 1 | 0.696 | 0.864 | 0.955 | 0.381 | 0.891 | 0.615 | 0.545 |
| 0 | 2 | 1 | 2 | 0.876 | 0.917 | 0.937 | 0.810 | 0.963 | 0.708 | 0.869 |
| 0 | 2 | 1 | 3 | 0.838 | 0.780 | 0.793 | 0.714 | 0.936 | 0.395 | 0.751 |
| 0 | 2 | 1 | 4 | 0.560 | 0.780 | 0.883 | 0.238 | 0.860 | 0.278 | 0.375 |
| 0 | 2 | 1 | 5 | 0.674 | 0.841 | 0.919 | 0.429 | 0.895 | 0.500 | 0.585 |
| 0 | 2 | 1 | 6 | 0.921 | 0.909 | 0.955 | 0.667 | 0.938 | 0.737 | 0.785 |
| 0 | 2 | 1 | 7 | 0.743 | 0.841 | 0.919 | 0.429 | 0.895 | 0.500 | 0.585 |
| 0 | 2 | 1 | 8 | 0.834 | 0.811 | 0.829 | 0.714 | 0.939 | 0.441 | 0.767 |
| 0 | 2 | 2 | 0 | 0.861 | 0.879 | 0.910 | 0.714 | 0.944 | 0.600 | 0.800 |
| 0 | 2 | 2 | 1 | 0.698 | 0.818 | 0.883 | 0.476 | 0.899 | 0.435 | 0.619 |
| 0 | 2 | 2 | 2 | 0.887 | 0.924 | 0.946 | 0.810 | 0.963 | 0.739 | 0.872 |
| 0 | 2 | 2 | 3 | 0.924 | 0.955 | 0.991 | 0.762 | 0.957 | 0.941 | 0.861 |
| 0 | 2 | 2 | 4 | 0.699 | 0.818 | 0.874 | 0.524 | 0.907 | 0.440 | 0.655 |
| 0 | 2 | 2 | 5 | 0.636 | 0.811 | 0.892 | 0.381 | 0.884 | 0.400 | 0.534 |
| 0 | 2 | 2 | 6 | 0.909 | 0.909 | 0.937 | 0.762 | 0.954 | 0.696 | 0.840 |
| 0 | 2 | 2 | 7 | 0.814 | 0.879 | 0.937 | 0.571 | 0.920 | 0.632 | 0.710 |
| 0 | 2 | 2 | 8 | 0.889 | 0.917 | 0.946 | 0.762 | 0.955 | 0.727 | 0.844 |
| 1 | 0 | 0 | 0 | 0.870 | 0.866 | 0.890 | 0.760 | 0.942 | 0.613 | 0.820 |
| 1 | 0 | 0 | 1 | 0.872 | 0.896 | 0.954 | 0.640 | 0.920 | 0.762 | 0.766 |
| 1 | 0 | 0 | 2 | 0.937 | 0.903 | 0.936 | 0.760 | 0.944 | 0.731 | 0.839 |
| 1 | 0 | 0 | 3 | 0.928 | 0.873 | 0.881 | 0.840 | 0.960 | 0.618 | 0.860 |
| 1 | 0 | 0 | 4 | 0.792 | 0.888 | 0.945 | 0.640 | 0.920 | 0.727 | 0.763 |
| 1 | 0 | 0 | 5 | 0.619 | 0.806 | 0.917 | 0.320 | 0.855 | 0.471 | 0.474 |
| 1 | 0 | 0 | 6 | 0.946 | 0.866 | 0.890 | 0.760 | 0.942 | 0.613 | 0.820 |
| 1 | 0 | 0 | 7 | 0.854 | 0.843 | 0.890 | 0.640 | 0.915 | 0.571 | 0.745 |
| 1 | 0 | 0 | 8 | 0.907 | 0.910 | 0.945 | 0.760 | 0.945 | 0.760 | 0.842 |
| 1 | 0 | 1 | 0 | 0.797 | 0.776 | 0.798 | 0.680 | 0.916 | 0.436 | 0.734 |
| 1 | 0 | 1 | 1 | 0.785 | 0.791 | 0.899 | 0.320 | 0.852 | 0.421 | 0.472 |
| 1 | 0 | 1 | 2 | 0.916 | 0.896 | 0.927 | 0.760 | 0.944 | 0.704 | 0.835 |
| 1 | 0 | 1 | 3 | 0.847 | 0.664 | 0.624 | 0.840 | 0.944 | 0.339 | 0.716 |
| 1 | 0 | 1 | 4 | 0.636 | 0.709 | 0.752 | 0.520 | 0.872 | 0.325 | 0.615 |
| 1 | 0 | 1 | 5 | 0.560 | 0.761 | 0.881 | 0.240 | 0.835 | 0.316 | 0.377 |
| 1 | 0 | 1 | 6 | 0.905 | 0.851 | 0.872 | 0.760 | 0.941 | 0.576 | 0.812 |
| 1 | 0 | 1 | 7 | 0.840 | 0.821 | 0.853 | 0.680 | 0.921 | 0.515 | 0.757 |
| 1 | 0 | 1 | 8 | 0.867 | 0.828 | 0.862 | 0.680 | 0.922 | 0.531 | 0.760 |
| 1 | 0 | 2 | 0 | 0.878 | 0.806 | 0.807 | 0.800 | 0.946 | 0.488 | 0.804 |
| 1 | 0 | 2 | 1 | 0.872 | 0.896 | 0.954 | 0.640 | 0.920 | 0.762 | 0.766 |
| 1 | 0 | 2 | 2 | 0.914 | 0.918 | 0.963 | 0.720 | 0.938 | 0.818 | 0.824 |
| 1 | 0 | 2 | 3 | 0.934 | 0.851 | 0.844 | 0.880 | 0.968 | 0.564 | 0.862 |
| 1 | 0 | 2 | 4 | 0.783 | 0.873 | 0.927 | 0.640 | 0.918 | 0.667 | 0.757 |
| 1 | 0 | 2 | 5 | 0.619 | 0.806 | 0.917 | 0.320 | 0.855 | 0.471 | 0.474 |
| 1 | 0 | 2 | 6 | 0.953 | 0.903 | 0.908 | 0.880 | 0.971 | 0.688 | 0.894 |
| 1 | 0 | 2 | 7 | 0.863 | 0.851 | 0.890 | 0.680 | 0.924 | 0.586 | 0.771 |
| 1 | 0 | 2 | 8 | 0.891 | 0.821 | 0.807 | 0.880 | 0.967 | 0.512 | 0.842 |
| 1 | 1 | 0 | 0 | 0.903 | 0.806 | 0.807 | 0.800 | 0.946 | 0.488 | 0.804 |
| 1 | 1 | 0 | 1 | 0.845 | 0.896 | 0.954 | 0.640 | 0.920 | 0.762 | 0.766 |
| 1 | 1 | 0 | 2 | 0.928 | 0.910 | 0.945 | 0.760 | 0.945 | 0.760 | 0.842 |
| 1 | 1 | 0 | 3 | 0.913 | 0.843 | 0.844 | 0.840 | 0.958 | 0.553 | 0.842 |
| 1 | 1 | 0 | 4 | 0.699 | 0.836 | 0.917 | 0.480 | 0.885 | 0.571 | 0.630 |
| 1 | 1 | 0 | 5 | 0.610 | 0.791 | 0.899 | 0.320 | 0.852 | 0.421 | 0.472 |
| 1 | 1 | 0 | 6 | 0.953 | 0.925 | 0.945 | 0.840 | 0.963 | 0.778 | 0.889 |
| 1 | 1 | 0 | 7 | 0.815 | 0.896 | 0.963 | 0.600 | 0.913 | 0.789 | 0.739 |
| 1 | 1 | 0 | 8 | 0.866 | 0.851 | 0.872 | 0.760 | 0.941 | 0.576 | 0.812 |
| 1 | 1 | 1 | 0 | 0.775 | 0.769 | 0.807 | 0.600 | 0.898 | 0.417 | 0.688 |
| 1 | 1 | 1 | 1 | 0.659 | 0.724 | 0.780 | 0.480 | 0.867 | 0.333 | 0.594 |
| 1 | 1 | 1 | 2 | 0.841 | 0.888 | 0.963 | 0.560 | 0.905 | 0.778 | 0.708 |
| 1 | 1 | 1 | 3 | 0.808 | 0.709 | 0.679 | 0.840 | 0.949 | 0.375 | 0.751 |
| 1 | 1 | 1 | 4 | 0.678 | 0.627 | 0.596 | 0.760 | 0.915 | 0.302 | 0.668 |
| 1 | 1 | 1 | 5 | 0.650 | 0.806 | 0.899 | 0.400 | 0.867 | 0.476 | 0.554 |
| 1 | 1 | 1 | 6 | 0.928 | 0.910 | 0.945 | 0.760 | 0.945 | 0.760 | 0.842 |
| 1 | 1 | 1 | 7 | 0.907 | 0.888 | 0.927 | 0.720 | 0.935 | 0.692 | 0.810 |
| 1 | 1 | 1 | 8 | 0.747 | 0.791 | 0.890 | 0.360 | 0.858 | 0.429 | 0.513 |
| 1 | 1 | 2 | 0 | 0.902 | 0.799 | 0.798 | 0.800 | 0.946 | 0.476 | 0.799 |
| 1 | 1 | 2 | 1 | 0.845 | 0.896 | 0.954 | 0.640 | 0.920 | 0.762 | 0.766 |
| 1 | 1 | 2 | 2 | 0.934 | 0.933 | 0.954 | 0.840 | 0.963 | 0.808 | 0.893 |
| 1 | 1 | 2 | 3 | 0.909 | 0.873 | 0.881 | 0.840 | 0.960 | 0.618 | 0.860 |
| 1 | 1 | 2 | 4 | 0.719 | 0.843 | 0.917 | 0.520 | 0.893 | 0.591 | 0.664 |
| 1 | 1 | 2 | 5 | 0.610 | 0.791 | 0.899 | 0.320 | 0.852 | 0.421 | 0.472 |
| 1 | 1 | 2 | 6 | 0.947 | 0.925 | 0.945 | 0.840 | 0.963 | 0.778 | 0.889 |
| 1 | 1 | 2 | 7 | 0.815 | 0.896 | 0.963 | 0.600 | 0.913 | 0.789 | 0.739 |
| 1 | 1 | 2 | 8 | 0.868 | 0.851 | 0.872 | 0.760 | 0.941 | 0.576 | 0.812 |
| 1 | 2 | 0 | 0 | 0.869 | 0.821 | 0.835 | 0.760 | 0.938 | 0.514 | 0.796 |
| 1 | 2 | 0 | 1 | 0.870 | 0.858 | 0.862 | 0.840 | 0.959 | 0.583 | 0.851 |
| 1 | 2 | 0 | 2 | 0.930 | 0.888 | 0.881 | 0.920 | 0.980 | 0.639 | 0.900 |
| 1 | 2 | 0 | 3 | 0.929 | 0.843 | 0.844 | 0.840 | 0.958 | 0.553 | 0.842 |
| 1 | 2 | 0 | 4 | 0.742 | 0.881 | 0.963 | 0.520 | 0.897 | 0.765 | 0.675 |
| 1 | 2 | 0 | 5 | 0.619 | 0.806 | 0.917 | 0.320 | 0.855 | 0.471 | 0.474 |
| 1 | 2 | 0 | 6 | 0.942 | 0.873 | 0.899 | 0.760 | 0.942 | 0.633 | 0.824 |
| 1 | 2 | 0 | 7 | 0.865 | 0.888 | 0.945 | 0.640 | 0.920 | 0.727 | 0.763 |
| 1 | 2 | 0 | 8 | 0.867 | 0.694 | 0.661 | 0.840 | 0.947 | 0.362 | 0.740 |
| 1 | 2 | 1 | 0 | 0.798 | 0.716 | 0.725 | 0.680 | 0.908 | 0.362 | 0.702 |
| 1 | 2 | 1 | 1 | 0.788 | 0.858 | 0.908 | 0.640 | 0.917 | 0.615 | 0.751 |
| 1 | 2 | 1 | 2 | 0.936 | 0.903 | 0.954 | 0.680 | 0.929 | 0.773 | 0.794 |
| 1 | 2 | 1 | 3 | 0.871 | 0.679 | 0.651 | 0.800 | 0.934 | 0.345 | 0.718 |
| 1 | 2 | 1 | 4 | 0.652 | 0.709 | 0.743 | 0.560 | 0.880 | 0.333 | 0.639 |
| 1 | 2 | 1 | 5 | 0.590 | 0.784 | 0.899 | 0.280 | 0.845 | 0.389 | 0.427 |
| 1 | 2 | 1 | 6 | 0.891 | 0.881 | 0.954 | 0.560 | 0.904 | 0.737 | 0.706 |
| 1 | 2 | 1 | 7 | 0.828 | 0.851 | 0.927 | 0.520 | 0.894 | 0.619 | 0.666 |
| 1 | 2 | 1 | 8 | 0.762 | 0.761 | 0.798 | 0.600 | 0.897 | 0.405 | 0.685 |
| 1 | 2 | 2 | 0 | 0.887 | 0.769 | 0.752 | 0.840 | 0.953 | 0.438 | 0.794 |
| 1 | 2 | 2 | 1 | 0.836 | 0.866 | 0.881 | 0.800 | 0.950 | 0.606 | 0.838 |
| 1 | 2 | 2 | 2 | 0.931 | 0.910 | 0.936 | 0.800 | 0.953 | 0.741 | 0.863 |
| 1 | 2 | 2 | 3 | 0.932 | 0.739 | 0.706 | 0.880 | 0.963 | 0.407 | 0.784 |
| 1 | 2 | 2 | 4 | 0.688 | 0.843 | 0.936 | 0.440 | 0.879 | 0.611 | 0.599 |
| 1 | 2 | 2 | 5 | 0.619 | 0.806 | 0.917 | 0.320 | 0.855 | 0.471 | 0.474 |
| 1 | 2 | 2 | 6 | 0.947 | 0.910 | 0.927 | 0.840 | 0.962 | 0.724 | 0.881 |
| 1 | 2 | 2 | 7 | 0.824 | 0.873 | 0.936 | 0.600 | 0.911 | 0.682 | 0.731 |
| 1 | 2 | 2 | 8 | 0.878 | 0.784 | 0.761 | 0.880 | 0.965 | 0.458 | 0.816 |
| 2 | 0 | 0 | 0 | 0.744 | 0.737 | 0.734 | 0.750 | 0.930 | 0.383 | 0.742 |
| 2 | 0 | 0 | 1 | 0.825 | 0.797 | 0.817 | 0.708 | 0.927 | 0.459 | 0.759 |
| 2 | 0 | 0 | 2 | 0.823 | 0.842 | 0.881 | 0.667 | 0.923 | 0.552 | 0.759 |
| 2 | 0 | 0 | 3 | 0.863 | 0.729 | 0.725 | 0.750 | 0.929 | 0.375 | 0.737 |
| 2 | 0 | 0 | 4 | 0.677 | 0.789 | 0.853 | 0.500 | 0.886 | 0.429 | 0.631 |
| 2 | 0 | 0 | 5 | 0.628 | 0.789 | 0.881 | 0.375 | 0.865 | 0.409 | 0.526 |
| 2 | 0 | 0 | 6 | 0.841 | 0.827 | 0.853 | 0.708 | 0.930 | 0.515 | 0.774 |
| 2 | 0 | 0 | 7 | 0.810 | 0.812 | 0.835 | 0.708 | 0.929 | 0.486 | 0.766 |
| 2 | 0 | 0 | 8 | 0.768 | 0.812 | 0.890 | 0.458 | 0.882 | 0.478 | 0.605 |
| 2 | 0 | 1 | 0 | 0.757 | 0.759 | 0.780 | 0.667 | 0.914 | 0.400 | 0.719 |
| 2 | 0 | 1 | 1 | 0.601 | 0.820 | 0.936 | 0.292 | 0.857 | 0.500 | 0.445 |
| 2 | 0 | 1 | 2 | 0.795 | 0.842 | 0.890 | 0.625 | 0.915 | 0.556 | 0.734 |
| 2 | 0 | 1 | 3 | 0.731 | 0.737 | 0.734 | 0.750 | 0.930 | 0.383 | 0.742 |
| 2 | 0 | 1 | 4 | 0.656 | 0.782 | 0.853 | 0.458 | 0.877 | 0.407 | 0.596 |
| 2 | 0 | 1 | 5 | 0.626 | 0.759 | 0.835 | 0.417 | 0.867 | 0.357 | 0.556 |
| 2 | 0 | 1 | 6 | 0.818 | 0.857 | 0.899 | 0.667 | 0.925 | 0.593 | 0.766 |
| 2 | 0 | 1 | 7 | 0.803 | 0.842 | 0.917 | 0.500 | 0.893 | 0.571 | 0.647 |
| 2 | 0 | 1 | 8 | 0.735 | 0.737 | 0.752 | 0.667 | 0.911 | 0.372 | 0.707 |
| 2 | 0 | 2 | 0 | 0.791 | 0.820 | 0.844 | 0.708 | 0.929 | 0.500 | 0.770 |
| 2 | 0 | 2 | 1 | 0.826 | 0.827 | 0.853 | 0.708 | 0.930 | 0.515 | 0.774 |
| 2 | 0 | 2 | 2 | 0.831 | 0.865 | 0.917 | 0.625 | 0.917 | 0.625 | 0.743 |
| 2 | 0 | 2 | 3 | 0.865 | 0.684 | 0.651 | 0.833 | 0.947 | 0.345 | 0.731 |
| 2 | 0 | 2 | 4 | 0.679 | 0.767 | 0.817 | 0.542 | 0.890 | 0.394 | 0.651 |
| 2 | 0 | 2 | 5 | 0.644 | 0.789 | 0.872 | 0.417 | 0.872 | 0.417 | 0.564 |
| 2 | 0 | 2 | 6 | 0.853 | 0.865 | 0.899 | 0.708 | 0.933 | 0.607 | 0.792 |
| 2 | 0 | 2 | 7 | 0.781 | 0.857 | 0.945 | 0.458 | 0.888 | 0.647 | 0.617 |
| 2 | 0 | 2 | 8 | 0.813 | 0.835 | 0.890 | 0.583 | 0.907 | 0.538 | 0.705 |
| 2 | 1 | 0 | 0 | 0.840 | 0.850 | 0.862 | 0.792 | 0.949 | 0.559 | 0.826 |
| 2 | 1 | 0 | 1 | 0.827 | 0.902 | 0.954 | 0.667 | 0.929 | 0.762 | 0.785 |
| 2 | 1 | 0 | 2 | 0.855 | 0.872 | 0.927 | 0.625 | 0.918 | 0.652 | 0.746 |
| 2 | 1 | 0 | 3 | 0.882 | 0.835 | 0.844 | 0.792 | 0.948 | 0.528 | 0.817 |
| 2 | 1 | 0 | 4 | 0.769 | 0.835 | 0.872 | 0.667 | 0.922 | 0.533 | 0.755 |
| 2 | 1 | 0 | 5 | 0.690 | 0.865 | 0.963 | 0.417 | 0.882 | 0.714 | 0.582 |
| 2 | 1 | 0 | 6 | 0.859 | 0.895 | 0.936 | 0.708 | 0.936 | 0.708 | 0.806 |
| 2 | 1 | 0 | 7 | 0.831 | 0.835 | 0.853 | 0.750 | 0.939 | 0.529 | 0.798 |
| 2 | 1 | 0 | 8 | 0.808 | 0.789 | 0.817 | 0.667 | 0.918 | 0.444 | 0.734 |
| 2 | 1 | 1 | 0 | 0.765 | 0.737 | 0.752 | 0.667 | 0.911 | 0.372 | 0.707 |
| 2 | 1 | 1 | 1 | 0.738 | 0.857 | 0.963 | 0.375 | 0.875 | 0.692 | 0.540 |
| 2 | 1 | 1 | 2 | 0.844 | 0.887 | 0.945 | 0.625 | 0.920 | 0.714 | 0.752 |
| 2 | 1 | 1 | 3 | 0.770 | 0.737 | 0.743 | 0.708 | 0.920 | 0.378 | 0.725 |
| 2 | 1 | 1 | 4 | 0.760 | 0.820 | 0.853 | 0.667 | 0.921 | 0.500 | 0.748 |
| 2 | 1 | 1 | 5 | 0.743 | 0.872 | 0.945 | 0.542 | 0.904 | 0.684 | 0.689 |
| 2 | 1 | 1 | 6 | 0.829 | 0.842 | 0.881 | 0.667 | 0.923 | 0.552 | 0.759 |
| 2 | 1 | 1 | 7 | 0.821 | 0.895 | 0.954 | 0.625 | 0.920 | 0.750 | 0.755 |
| 2 | 1 | 1 | 8 | 0.747 | 0.797 | 0.862 | 0.500 | 0.887 | 0.444 | 0.633 |
| 2 | 1 | 2 | 0 | 0.840 | 0.842 | 0.853 | 0.792 | 0.949 | 0.543 | 0.821 |
| 2 | 1 | 2 | 1 | 0.827 | 0.902 | 0.954 | 0.667 | 0.929 | 0.762 | 0.785 |
| 2 | 1 | 2 | 2 | 0.827 | 0.895 | 0.936 | 0.708 | 0.936 | 0.708 | 0.806 |
| 2 | 1 | 2 | 3 | 0.880 | 0.872 | 0.890 | 0.792 | 0.951 | 0.613 | 0.838 |
| 2 | 1 | 2 | 4 | 0.748 | 0.827 | 0.872 | 0.625 | 0.913 | 0.517 | 0.728 |
| 2 | 1 | 2 | 5 | 0.715 | 0.880 | 0.972 | 0.458 | 0.891 | 0.786 | 0.623 |
| 2 | 1 | 2 | 6 | 0.849 | 0.865 | 0.917 | 0.625 | 0.917 | 0.625 | 0.743 |
| 2 | 1 | 2 | 7 | 0.848 | 0.872 | 0.917 | 0.667 | 0.926 | 0.640 | 0.772 |
| 2 | 1 | 2 | 8 | 0.812 | 0.835 | 0.890 | 0.583 | 0.907 | 0.538 | 0.705 |
| 2 | 2 | 0 | 0 | 0.836 | 0.835 | 0.853 | 0.750 | 0.939 | 0.529 | 0.798 |
| 2 | 2 | 0 | 1 | 0.784 | 0.835 | 0.899 | 0.542 | 0.899 | 0.542 | 0.676 |
| 2 | 2 | 0 | 2 | 0.823 | 0.880 | 0.945 | 0.583 | 0.912 | 0.700 | 0.721 |
| 2 | 2 | 0 | 3 | 0.864 | 0.759 | 0.761 | 0.750 | 0.933 | 0.409 | 0.756 |
| 2 | 2 | 0 | 4 | 0.723 | 0.812 | 0.862 | 0.583 | 0.904 | 0.483 | 0.696 |
| 2 | 2 | 0 | 5 | 0.612 | 0.789 | 0.890 | 0.333 | 0.858 | 0.400 | 0.485 |
| 2 | 2 | 0 | 6 | 0.856 | 0.895 | 0.945 | 0.667 | 0.928 | 0.727 | 0.782 |
| 2 | 2 | 0 | 7 | 0.828 | 0.865 | 0.927 | 0.583 | 0.910 | 0.636 | 0.716 |
| 2 | 2 | 0 | 8 | 0.788 | 0.729 | 0.743 | 0.667 | 0.910 | 0.364 | 0.703 |
| 2 | 2 | 1 | 0 | 0.755 | 0.707 | 0.706 | 0.708 | 0.917 | 0.347 | 0.707 |
| 2 | 2 | 1 | 1 | 0.701 | 0.812 | 0.899 | 0.417 | 0.875 | 0.476 | 0.569 |
| 2 | 2 | 1 | 2 | 0.799 | 0.835 | 0.908 | 0.500 | 0.892 | 0.545 | 0.645 |
| 2 | 2 | 1 | 3 | 0.786 | 0.699 | 0.679 | 0.792 | 0.937 | 0.352 | 0.731 |
| 2 | 2 | 1 | 4 | 0.688 | 0.782 | 0.835 | 0.542 | 0.892 | 0.419 | 0.657 |
| 2 | 2 | 1 | 5 | 0.658 | 0.812 | 0.899 | 0.417 | 0.875 | 0.476 | 0.569 |
| 2 | 2 | 1 | 6 | 0.838 | 0.774 | 0.798 | 0.667 | 0.916 | 0.421 | 0.727 |
| 2 | 2 | 1 | 7 | 0.798 | 0.812 | 0.890 | 0.458 | 0.882 | 0.478 | 0.605 |
| 2 | 2 | 1 | 8 | 0.756 | 0.654 | 0.624 | 0.792 | 0.932 | 0.317 | 0.698 |
| 2 | 2 | 2 | 0 | 0.821 | 0.842 | 0.853 | 0.792 | 0.949 | 0.543 | 0.821 |
| 2 | 2 | 2 | 1 | 0.784 | 0.835 | 0.899 | 0.542 | 0.899 | 0.542 | 0.676 |
| 2 | 2 | 2 | 2 | 0.886 | 0.872 | 0.917 | 0.667 | 0.926 | 0.640 | 0.772 |
| 2 | 2 | 2 | 3 | 0.845 | 0.835 | 0.853 | 0.750 | 0.939 | 0.529 | 0.798 |
| 2 | 2 | 2 | 4 | 0.723 | 0.812 | 0.862 | 0.583 | 0.904 | 0.483 | 0.696 |
| 2 | 2 | 2 | 5 | 0.612 | 0.789 | 0.890 | 0.333 | 0.858 | 0.400 | 0.485 |
| 2 | 2 | 2 | 6 | 0.861 | 0.910 | 0.954 | 0.708 | 0.937 | 0.773 | 0.813 |
| 2 | 2 | 2 | 7 | 0.828 | 0.865 | 0.927 | 0.583 | 0.910 | 0.636 | 0.716 |
| 2 | 2 | 2 | 8 | 0.772 | 0.737 | 0.743 | 0.708 | 0.920 | 0.378 | 0.725 |
